# *In-silico* Investigation of Antitrypanosomal Phytochemicals from Nigerian Medicinal Plants

**DOI:** 10.1371/journal.pntd.0001727

**Published:** 2012-07-24

**Authors:** William N. Setzer, Ifedayo V. Ogungbe

**Affiliations:** 1 Department of Chemistry, University of Alabama in Huntsville, Huntsville, Alabama, United States of America; 2 Department of Metabolism and Aging, Scripps Research Institute, Jupiter, Florida, United States of America; University of Tokyo, Japan

## Abstract

**Background:**

Human African trypanosomiasis (HAT), a parasitic protozoal disease, is caused primarily by two subspecies of *Trypanosoma brucei*. HAT is a re-emerging disease and currently threatens millions of people in sub-Saharan Africa. Many affected people live in remote areas with limited access to health services and, therefore, rely on traditional herbal medicines for treatment.

**Methods:**

A molecular docking study has been carried out on phytochemical agents that have been previously isolated and characterized from Nigerian medicinal plants, either known to be used ethnopharmacologically to treat parasitic infections or known to have *in-vitro* antitrypanosomal activity. A total of 386 compounds from 19 species of medicinal plants were investigated using *in-silico* molecular docking with validated *Trypanosoma brucei* protein targets that were available from the Protein Data Bank (PDB): Adenosine kinase (TbAK), pteridine reductase 1 (TbPTR1), dihydrofolate reductase (TbDHFR), trypanothione reductase (TbTR), cathepsin B (TbCatB), heat shock protein 90 (TbHSP90), sterol 14α-demethylase (TbCYP51), nucleoside hydrolase (TbNH), triose phosphate isomerase (TbTIM), nucleoside 2-deoxyribosyltransferase (TbNDRT), UDP-galactose 4′ epimerase (TbUDPGE), and ornithine decarboxylase (TbODC).

**Results:**

This study revealed that triterpenoid and steroid ligands were largely selective for sterol 14α-demethylase; anthraquinones, xanthones, and berberine alkaloids docked strongly to pteridine reductase 1 (TbPTR1); chromenes, pyrazole and pyridine alkaloids preferred docking to triose phosphate isomerase (TbTIM); and numerous indole alkaloids showed notable docking energies with UDP-galactose 4′ epimerase (TbUDPGE). Polyphenolic compounds such as flavonoid gallates or flavonoid glycosides tended to be promiscuous docking agents, giving strong docking energies with most proteins.

**Conclusions:**

This *in-silico* molecular docking study has identified potential biomolecular targets of phytochemical components of antitrypanosomal plants and has determined which phytochemical classes and structural manifolds likely target trypanosomal enzymes. The results could provide the framework for synthetic modification of bioactive phytochemicals, *de novo* synthesis of structural motifs, and lead to further phytochemical investigations.

## Author Summary

Traditional herbal medicine continues to play a key role in health, particularly in remote areas with limited access to “modern medicines”. Many plants are used in traditional Nigerian medicine to treat parasitic diseases. While many of these plants have shown notable activity against parasitic protozoa, in most cases the mode of activity is not known. That is, it is not known what biochemical entities are being targeted by the plant chemical constituents. In this work, we have carried out molecular docking studies of known phytochemicals from Nigerian medicinal plants used to treat human African trypanosomiasis (sleeping sickness) with known biochemical targets in the *Trypanosoma brucei* parasite. The goals of this study were to identify the protein targets that the medicinal plants are affecting and to discern general trends in protein target selectivity for phytochemical classes. In doing so, we have theoretically identified strongly interacting plant chemicals and their biomolecular targets. These results should lead to further research to verify the efficacy of the phytochemical agents as well as delineate possible modifications of the active compounds to increase potency or selectivity.

## Introduction

Human African trypanosomiasis (HAT), also known as *sleeping sickness*, is caused by the single-celled kinetoplastid parasites, *Trypanosoma brucei*, which are transmitted to humans by infected tsetse flies (*Glossina* spp.). Two sub-species of *T. brucei* (*rhodesiense* and *gambiense*) cause the two different forms of the disease. *T. b. rhodesiense* is found in southern and eastern Africa while *T. b. gambiense* is found in the western, central and some parts of eastern Africa. *T. b. gambiense* now accounts for about 90% of all reported cases of sleeping sickness. A third subspecies, *T. b. brucei*, does not cause HAT because of its susceptibility to lysis by human apolipoprotein L1 [1].

Current chemotherapies of HAT are directed either to the early or late stages of the disease. All the clinically available HAT chemotherapeutic drugs have been noted to be ineffective, and they also have severe side-effects. The only drug candidate in clinical trials for the treatment of HAT is the nitroimidazole fexinidazole. Fexinidazole is currently in clinical study for the treatment of the late stage form of HAT [2], [3]. It is worth noting that the number of reported cases of HAT fell in the past decade, and it has also been suggested that a possible elimination of the disease might be in sight [4]. This is a very delightful development for this “neglected” tropical disease, and it is our hope that continued research into new and effective chemotherapy against HAT remains an integral part of public health initiatives in endemic communities.

Medicinal plants from Nigeria's lush rainforest, as well as her very diverse montane and savanna vegetation, continue to play a vital role in her healthcare system. For tens of millions of Nigerians, indigenous traditional medicine is the major – and sometimes the only – access to pharmacological agents [5]. There have been several published reports on the biological activity of Nigerian plants, but most of the bioactive components of those plants have not been characterized. However, the country's big and loosely-regulated traditional medicine industry continues to promote the efficacy of extracts and concoctions made from most of the plants. A number of Nigerian plants have been used traditionally in West Africa to treat protozoal infections and many of these have shown *in-vitro* antiprotozoal activity (Table S1).

Several *T. brucei* protein targets have been identified and experimentally validated [6]. In addition to validated targets, several potential targets have been predicted *in silico* [7]. For a recent review of phytochemical agents that show activities against parasitic protozoans and protozoan biochemical targets, see [8], [9]. Some of the potential *T. brucei* drug targets that we considered in this work include adenosine kinase [10], pteridine reductase 1 [11], dihydrofolate reductase [12], trypanothione reductase [13], cathepsin B [14], heat shock protein 90 [15], as well as sterol 14α-demethylase (CYP51) [16], nucleoside hydrolase [17], triose phosphate isomerase [18], nucleoside 2-deoxyribosyltransferase [19], UDP-galactose 4′ epimerase [20] and ornithine decarboxylase [21]. In this computational study, we have evaluated the interaction of compounds that were isolated from some antitrypanosomal Nigerian medicinal plants (Table S1) against potential protein drug targets in *Trypanosoma brucei* for which X-ray crystal structures were available from the Protein Data Bank (PDB). We strove to address the questions of which phytochemical agents might be responsible for the observed antitrypanosomal activity and what are the likely targets of those phytochemicals. In doing so, we hope to identify particular classes of phytochemical agents that can be exploited for antiparasitic chemotherapy.

## Methods

Protein-ligand docking studies were carried out based on the crystal structures of rhodesain (PDB 2p7u, [22] and PDB 2p86 [23]), *T. brucei* adenosine kinase, TbAK (PDB 2xtb and PDB 3otx [24]), *T. brucei* pteridine reductase 1, TbPTR1 (PDB 3jq7 [25]), *T. brucei* dihydrofolate reductase, TbDHFR (PDB 3rg9 and PDB 3qfx [26]), *T. brucei* trypanothione reductase, TbTR (PDB 2wow, [27]), *T. brucei* cathepsin B, TbCatB (PDB 3hhi [28]), *T. brucei* heat shock protein 90, TbHSP90 (PDB 3omu [29] and PDB 3opd [30]), *T. brucei* sterol 14α-demethylase, TbCYP51 (PDB 3gw9 [16]), *T. brucei* nucleoside hydrolase, TbNH (PDB 3fz0 [31]), *T. brucei* triosephosphate isomerase, TbTIM (PDB 1iih, PDB 6tim [32], and PDB 4tim [33]), *T. brucei* nucleoside 2-deoxyribosyltransferase, TbNDRT (PDB 2a0k, PDB 2f64, and PDB 2f67 [19]), *T. brucei* UDP-galactose 4*′*-epimerase, TbUDPGE (PDB 1gy8 [20]), and *T. brucei* ornithine decarboxylase, TbODC (PDB 1f3t [34], PDB 1njj [35], and PDB 1qu4 [21]). All solvent molecules and the co-crystallized ligands were removed from the structures. Molecular docking calculations for all compounds with each of the proteins were undertaken using Molegro Virtual Docker v. 4.3 [36], [37], with a sphere large enough to accommodate the cavity centered on the binding sites of each protein structure in order to allow each ligand to search. If a co-crystallized inhibitor or substrate was present in the structure, then that site was chosen as the binding site. If no co-crystallized ligand was present, then suitably sized cavities were used as potential binding sites. Standard protonation states of the proteins based on neutral pH were used in the docking studies. The protein was used as a rigid model structure; no relaxation of the protein was performed. Assignments of charges on each protein were based on standard templates as part of the Molegro Virtual Docker program; no other charges were set. Each ligand structure was built using Spartan '08 for Windows [38]. The structures were geometry optimized using the MMFF force field [39]. Flexible ligand models were used in the docking and subsequent optimization scheme. As a test of docking accuracy and for docking energy comparison, co-crystallized ligands were re-docked into the protein structures. Different orientations of the ligands were searched and ranked based on their energy scores. The RMSD threshold for multiple cluster poses was set at <1.00 Å. The docking algorithm was set at maximum iterations of 1500 with a simplex evolution population size of 50 and a minimum of 30 runs for each ligand. Each binding site of oligomeric structures was searched with each ligand. The lowest-energy (strongest-docking) poses for each ligand in each protein target are summarized in Tables S2–S20.

## Results and Discussion

### Acacia nilotica

Phytochemical studies of *Acacia nilotica* [40]–[45] have shown an abundance of polyphenolic compounds (Table S2), including hydrolyzable tannins, flavonoid gallates, and flavonoid glycosides. Although these polyphenolics are notorious for being promiscuous protein complexing agents and they do show relatively strong docking to all proteins investigated in this study, some selectivity can be seen. Thus, for example, 1,3-digalloylglucose showed docking selectivity for TbUDPGE, 3*′*,5-digalloylcatechin was selective for TbAK, and 3*′*,7-digalloylcatechin selectively docked with TbNH and was the strongest binding ligand for that protein (−44.2 kcal/mol). 5,7-Digalloylcatechin was the strongest binding ligand for TbPTR1 (−42.7 kcal/mol) and 4*′*,7-digalloylcatechin was the strongest binding ligand for TbODC (−41.4 kcal/mol). A number of these polyphenolic ligands showed strong docking interactions with TbAK, TbPTR1, TbCYP51, TbNH, and TbUDPGE, and interactions with these protein targets may be responsible for the antitrypanosomal activity of *A. nilotica* [46]. The docking study suggests that rhodesain, TbDHFR, TbTR, TbCatB, and TbHSP90 are not targets for *A. nilotica* phytochemicals.

### Ageratum conyzoides

*Ageratum conyzoides* extracts have been dominated by flavonoids and chromanes (Table S3) [40], [47]–[49]. 5,6-Dimethoxy-2-isopropylbenzofuran, 6,7-dimethoxy-2-methyl-2-(2-methyl-1-propanone)-3-chromene, 6-acetyl-2,2-dimethylchroman, and *O*-methylenececalinol exhibited selectivity for TbTIM with docking energies comparable to the co-crystallized ligand, 3-phosphoglyceric acid (−21.6 kcal/mol). The flavonoid 3*′*,4*′*,5,5*′*,6,8-hexamethoxyflavone, on the other hand, showed selective docking to TbPTR1 and TbUDPGE. Nour and co-workers [49] have examined the antitrypanosomal activities of several methylated flavonoids and a chromene from *A. conyzoides*. The flavonoids all have similar antitrypanosomal activities with *IC*_50_ values ranging from 3.0 to 6.7 µg/mL. The chromene, *O*-methylencedalinol, on the other hand, was much less active (*IC*_50_ = 78.4 µg/mL). The docking energies for many of the protein targets was much more negative (stronger docking) for the flavonoids than for the chromene. Thus, for example, there is good correlation between log(*IC*_50_) and docking energies of the ligands with TbPTR1 or with TbUDPGE (*R* ^2^ = 0.712 and 0.751, respectively).

### Annona senegalensis

Compounds isolated from *Annona senegalensis* include annonaceous acetogenins, diterpenoids, and sesquiterpenoids, and aporphine alkaloids (Table S4) [40], [50]–[53]. The acetogenins (annogalene, annonacin, annonacin A, annosenegalin, and senegalene) are probably responsible for the antitrypanosomal activity of the plant [54], [55]. These compounds show a propensity for docking with TbAK, TbCYP51, and TbUDPGE. The acetogenins are very flexible with a great deal of conformational mobility. Nevertheless, docking with these protein targets is largely hydrophobic. Key interactions of the acetogenins with TbAK include Phe337, Gly298, Asn295, Asn67, and Gly296. Additionally, the acetogenin annogalene is one of the best binding ligands for TbDPGE (−42.9 kcal/mol).

### Bridelia ferruginea

*Bridelia ferruginea* has been phytochemically characterized with polyphenolic and triterpenoid constituents (Table S5) [40], [56]. The flavonoids delphinidin and ferrugin showed docking selectivity for TbPTR1. The tannin epigallocatechin(7→4′)gallocatechin showed notably strong docking with TbCYP51. Although they are relatively weak docking ligands, the triterpenoids friedelin and taraxerol docked selectively with TbUDPGE.

### Carapa procera

**Figure 1:**
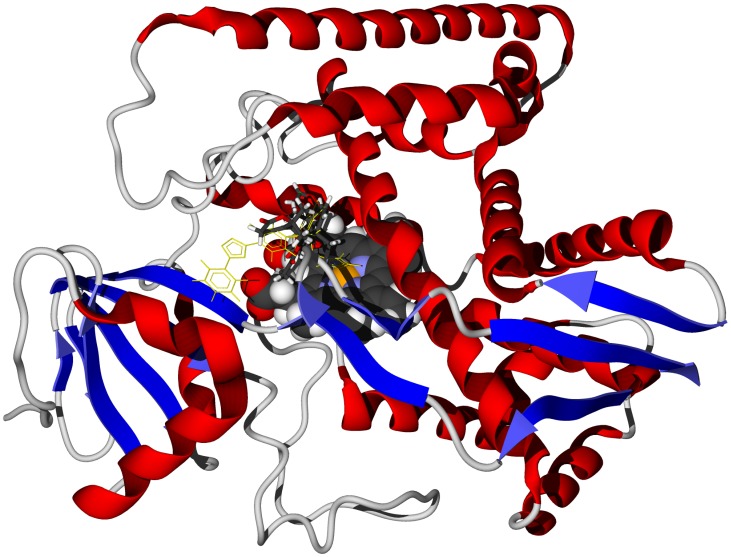
The crystal structure of *T. brucei* sterol 14α-demethylase, TbCYP51 (PDB 3gw9) [34]. The docked ligand is carapolide A (stick figure). The co-crystallized ligand is shown as a green wire figure and the heme cofactor is shown as a space-filling structure.

Limonoids are characteristic phytochemicals of the Meliaceae, including *Carapa procera* (Table S6) [40], [57], and numerous limonoids have exhibited antiprotozoal activities [58]–[62]. Six of the eleven *C. procera* limonoids showed notably strong docking with TbCYP51 (docking energies<−26 kcal/mol). A similar trend was noted for docking of *Khaya* limonoids (see below). Carapolides A, B, and C showed particularly strong docking with docking energies of −31.8, −29.3, and −28.5 kcal/mol, respectively; comparable to the docking energy of the co-crystallized ligand, *N*-[(1*R*)-1-(2,4-dichlorophenyl)-2-(1*H*-imidazol-1-yl)ethyl]-4-(5-phenyl-1,3,4-oxadiazol-2-yl)benzamide [16] (−28.6 kcal/mol), for this protein. The limonoids all dock with TbCYP51 near the heme cofactor (Fig. 1). In addition, preferential docking of individual limonoids with other protein targets include: mexicanolide with TbAK, 3β-isobutyroloxy-1-oxomeliac-8(30)-enate with TbPTR1, and evodulone with TbCatB. We conclude, therefore, that *T. brucei* sterol 14α-demethylase, TbCYP51, is a protein target of *C. procera* limonoids.

### Enantia chlorantha

**Figure 2:**
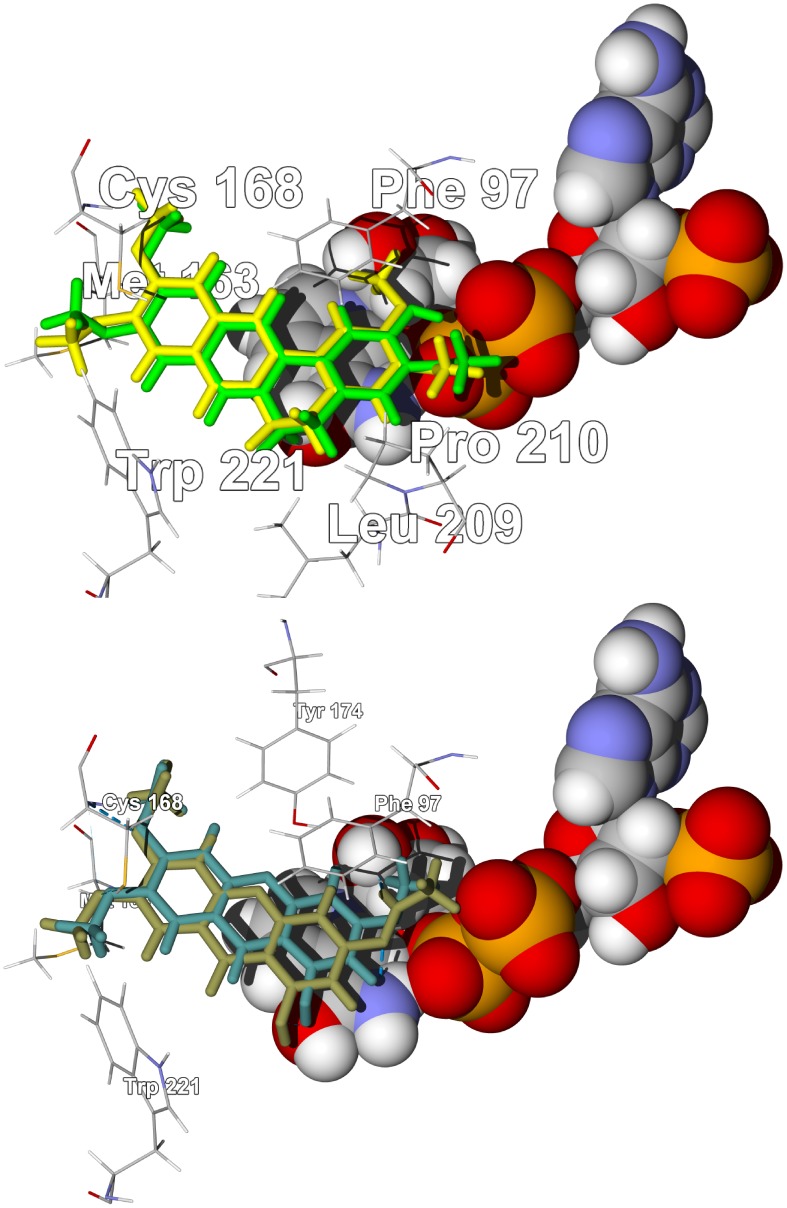
The crystal structure of *T. brucei* pteridine reductase 1, TbPTR1 (PDB 3jq7) [**43**]. Top: Lowest-energy docking poses of pseudocolumbamine (green stick figure) and pseudopalmatine (yellow stick figure) in the crystal structure. The NADP^+^ cofactor is shown as a space-filling structure. Bottom: Lowest-energy docking poses of laxanthone II (brown stick figure) and laxanthone III (dark green stick figure) in the same crystal structure.

*Enantia chlorantha* is dominated by aporphine and berberine alkaloids (Table S7) [40], [63], [64]. *E. chlorantha* aporphine alkaloids seem to show a propensity for docking with TbPTR1 or with TbUDPGE while the berberine alkaloids showed selectivity for TbPTR1. Both pseudocolumbamine and pseudopalmatine docked with TbPTR1 with docking energies (−27.5 kcal/mol) comparable to the co-crystallized ligand, 6-phenylpteridine-2,4,7-triamine [25] (−27.6 kcal/mol). These planar alkaloids dock into the active site by way of hydrophobic interactions with the NADP^+^ cofactor and a hydrophobic pocket formed by Phe97, Met163, Cys168, Pro210, Trp221, and Leu209 (Fig. 2). Liriodenine and columbamine docked selectively to TbTIM with docking energies lower (−24.0 and −24.7 kcal/mol) than the co-crystallized ligand, 3-phosphoglyceric acid [32] (−21.6 kcal/mol). These nearly planar alkaloids are known also to be DNA intercalators and topoisomerase inhibitors [65].

### Garcinia kola

**Figure 3:**
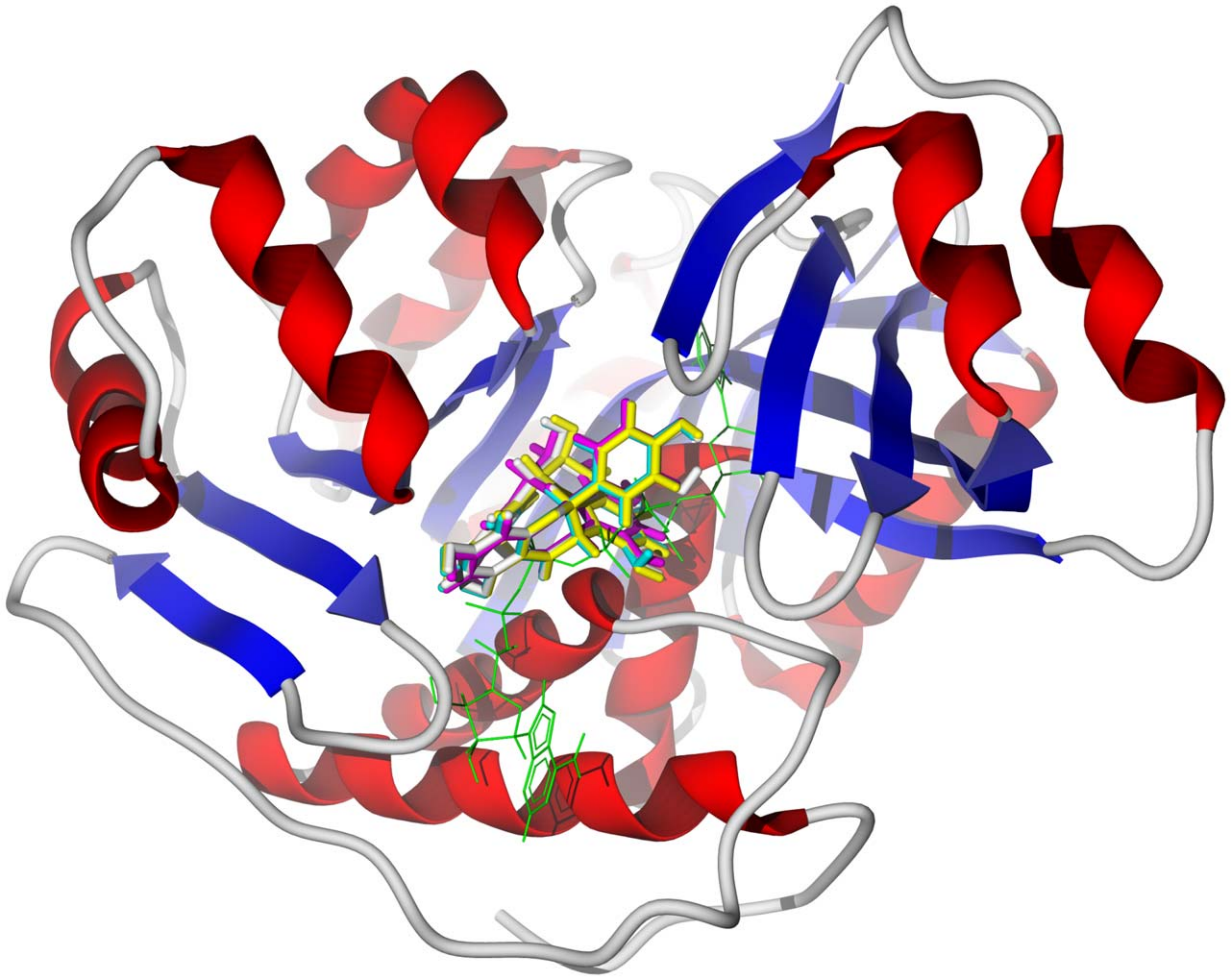
The crystal structure of *T. brucei* adenosine kinase, TbAK (PDB 3otx) [**42**]. The docked poses are the biflavonoids GB1 (turquoise), GB1a (magenta), GB2 (yellow), and garciniflavanone (white). The co-crystallized ligand, *bis*(adenosine)-5*′*-pentaphosphate, is shown as a green wire figure.

**Figure 4:**
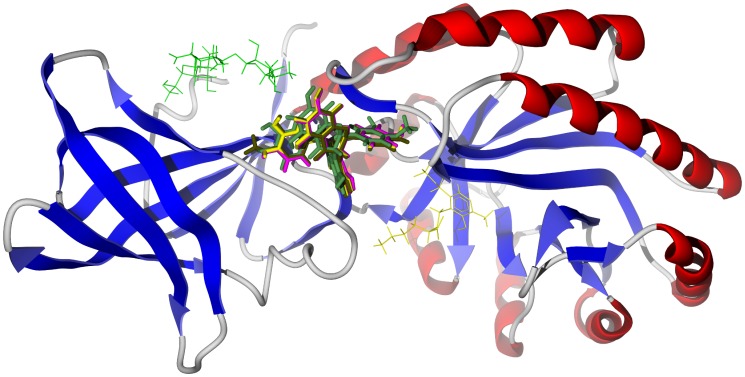
The crystal structure of *T. brucei* ornithine decarboxylase, TbODC (PDB 1njj) [**53**]. The docked poses are the biflavonoids GB1a (magenta), GB2 (yellow), GB3 (green) and kolaflavanone (brown). The co-crystallized ligands are geneticin (green wire figure) and pyridoxylphosphate/d-ornithine (yellow wire figure).

**Figure 5:**
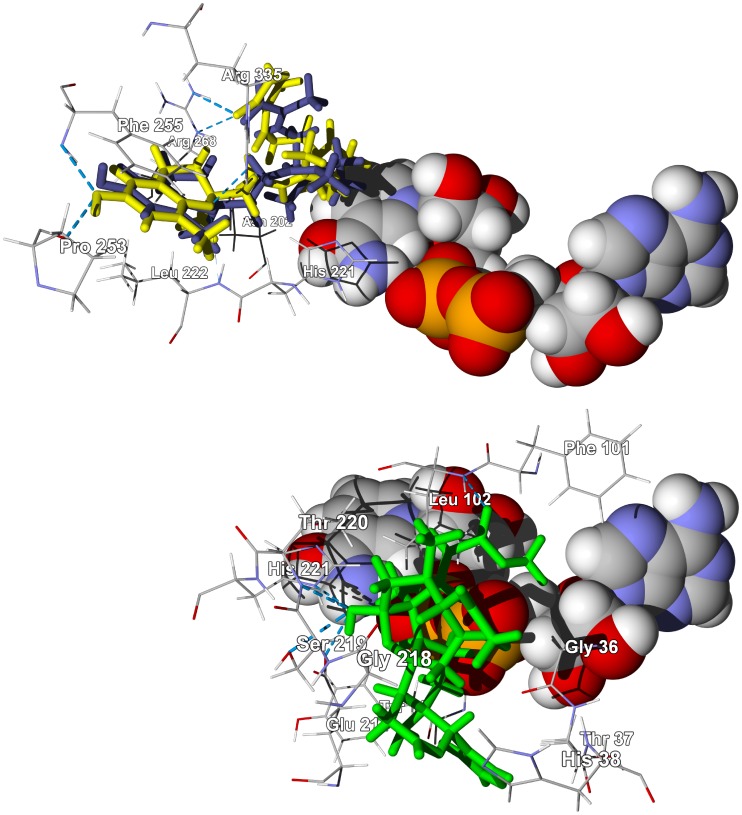
The crystal structure of *T. brucei* UDP-galactose 4*′*-epimerase, TbUDPGE (PDB 1gy8) [**37**]. Top: Lowest-energy docked poses of garcinal (purple stick figure) and garcinoic acid (yellow stick figure) showing key hydrogen-bonding and hydrophobic interactions. The NAD cofactor is shown as a space-filling structure; hydrogen bonds are depicted as blue dashed lines. Bottom: Lowest-energy docked pose of 3-*O*-acetylkhayalactone (green stick figure) in the same crystal structure.

**Figure 6:**
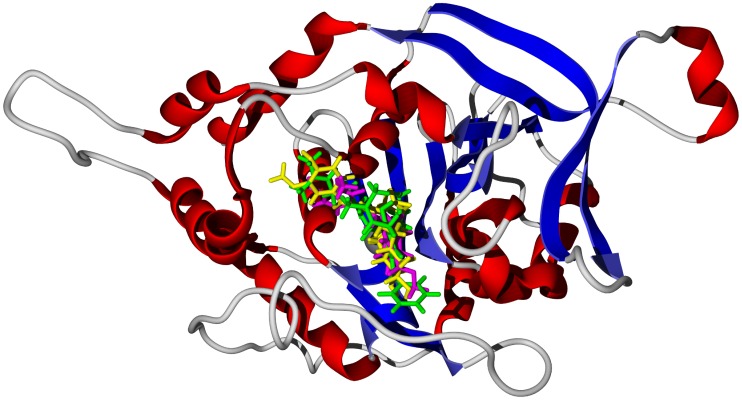
The X-ray crystal structure of *T. brucei* nucleoside hydrolase, Tb NH (PDB 3fz0) [**49**]. The lowest energy docking poses of kolanone (green), oruwacin (magenta), and dehydroepoxymethoxygaertneroside (yellow) are shown in the active site.

Polyphenolic compounds, flavonoids, biflavonoids, etc., have been isolated and identified from *Garcinia kola* (Table S8) [40], [66]. *G. kola* biflavonoids docked favorably with TbAK and TbODC. The biflavonoids do not dock at the adenosine binding sites of TbAK, but rather in a pocket between the two sites bounded by residues Asn222, Gly298, Ala297, Thr264, Asp266, Glu225, Arg132, and Asn195 (see Fig. 3). Likewise, biflavonoid docking with TbODC does not occur at the ornithine/putrescine binding site or the geneticin binding site, but rather in a pocket bounded by Asp243, Asp385, Val335, Asp332, Ala334, Ala244, and Arg277 (Fig. 4). This would suggest that if *G. kola* biflavonoids inhibit either TbAK or TbODC, they act as allosteric inhibitors of these proteins. The two tocotrienols garcinal and garcinoic acid, on the other hand, docked more favorably with TbUDPGE. Key interactions of the tocotrienols with the protein are hydrogen-bonding of the phenolic –OH of the ligands with Pro253 and Phe255, hydrogen-bonding of the carbonyl group of the ligand side chains with Arg268, hydrogen-bonding of the pyran ring oxygen atom with Arg235, face-to-face π – π interactions of the ligand aromatic rings with Phe255, and hydrophobic interactions of the tocotrienol ligands with Leu222, His221 and the NAD cofactor (Fig. 5 top). The prenylated benzophenone kolanone docked very strongly with TbNH (docking energy = −37.1 kcal/mol) in the nucleoside binding site (Fig. 6), a hydrophobic pocket bounded by Trp80, Phe178, Asn171, Trp205, and Val277, with additional hydrogen-bonding with Asn171.

### Khaya ivorensis and Khaya senegalensis

**Figure 7:**
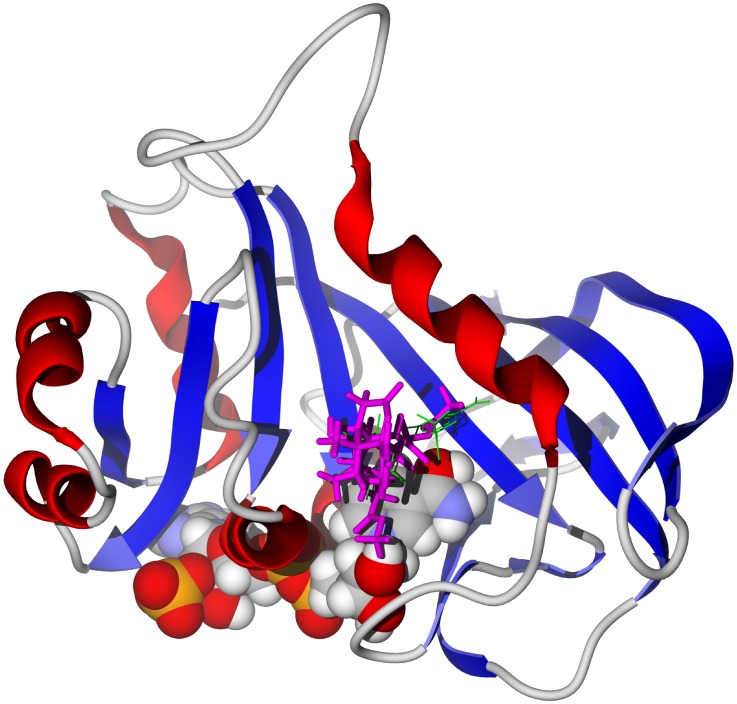
The crystal structure of *T. brucei* dihydrofolate reductase, TbDHFR (PDB 3qfx) [**44**]. The docked structure is 3-*O*-acetylkhayalactone (magenta). The co-crystallized ligand, pyrimethamine, is shown as a green wire figure and the NADPH cofactor as a space-filling structure.

The phytochemical compositions of *Khaya ivorensis* [67] and *K. senegalensis* [58], [68]–[72], like other members of the Meliaceae, are characterized by limonoids [40]. Many of the *Khaya* limonoids showed markedly strong docking to TbAK as well as TbCYP51 (see Table S9). Of particular note, 3-*O*-acetylkhayalactone strongly docked with TbAK, TbDHFR, and TbUDPGE (−31.6, −32.2, and −34.2 kcal/mol, respectively). This ligand docked in the same site in TbAK as the *Garcinia* biflavonoids (above), but in a different position in TbUDPGE (Fig. 5 bottom). Important hydrogen-bonding interactions of 3-*O*-acetylkhayalactone with TbUDPGE are with residues Glu214, Ser219, Leu102, Thr220, and His221. 3-*O*-Acetylkhayalactone docked in the active site of TbDHFR in the same general location as the co-crystallized ligand (Fig. 7). In addition, the docking energy of 3-*O*-acetylkhayalactone (−32.2 kcal/mol) was lower than either of the co-crystallized ligands, 5-(4-chlorophenyl)-6-ethylpyrimidine-2,4-diamine (pyrimethamine) and 6,6-dimethyl-1-[3-(2,4,5-trichlorophenoxy)propoxy]-1,6-dihydro-1,3,5-triazine-2,4-diamine [26] (−22.7 and −30.1 kcal/mol, respectively). In general, the *Khaya* limonoids showed weak or no docking with TbNH, TbTIM, or TbNDRT.

### Lawsonia inermis

The sterols and triterpenoids [40] from *Lawsonia inermis* showed preferential docking to TbCYP51 (*T. brucei* sterol 14α-demethylase) (Table S10). This is perhaps not surprising since the normal substrates for this enzyme are sterols. The laxanthones from *L. inermis* showed preferential docking to TbPTR1 with docking energies comparable to the co-crystallized ligand. In addition, they docked in the same positions and orientations as pseudocolumbamine and pseudopalmatine from *Enantia chlorantha* (see above and Fig. 2).

### Morinda lucida

**Figure 8:**
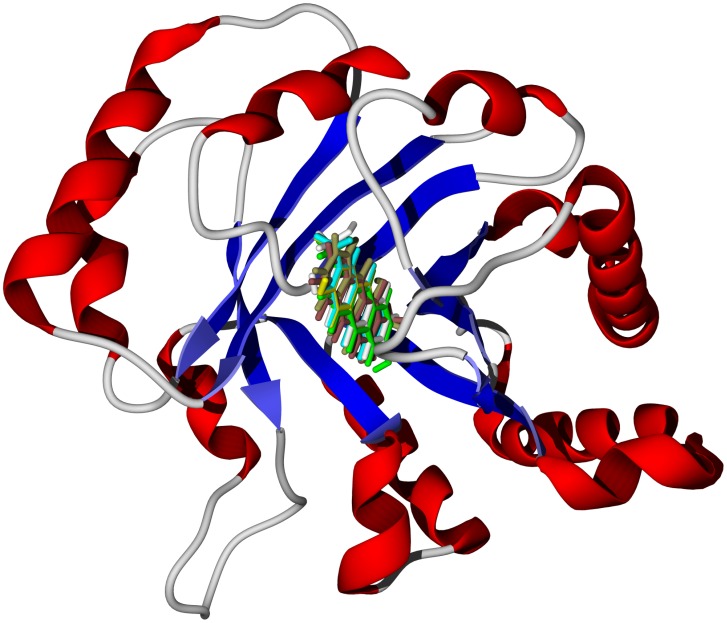
The crystal structure of *T. brucei* triosephosphate isomerase, TbTIM (PDB 1iih) [**50**]. The docked structures are the lowest-energy docking poses of the strongly docking *M. lucida* anthraquinones (2-formyl-3-hydroxyanthaquinone, 2-formylanthraquinone, 2-hydroxy-3-hydroxymethyl-anthraquinone, nordamnacanthal, rubiadin, and soranjidiol) in the active site of the protein.

Anthraquinones dominate the phytochemistry of *Morinda lucida* [40], [73], [74], along with triterpenoid acids [75] (see Table S11). Anthraquinones, as a class, demonstrated significant docking affinity for TbPRT1 and TbTIM (Fig. 8). Anthraquinones are also known to be DNA intercalators and topoisomerase inhibitors [76]. The triterpenoid acids oleanolic acid and ursolic acid showed notable docking energies with TbCYP51 (see above). The phenylpropanoid oruwacin docked very strongly to TbPTR1 (docking energy = −32.4 kcal/mol) and TbNH (docking energy = −31.6 kcal/mol, Fig. 6).

### Morinda morindoides

The phytochemistry of *Morinda morindoides* [40] is dominated by flavonoid glycosides [77] and phenylpropanoid-conjugated iridoid glycosides [78] (Table S12). Of these, epoxygaertneroside and morindaoside were selectively strongly binding ligands for TbAK, and morindaoside also docked strongly to TbPTR1. A number of gaertneroside derivatives showed docking selectivity for TbDHFR (see Table S12), while dehydroepoxymethoxygaertneroside docked very strongly with TbNH, occupying the nucleoside binding site (Fig. 6) with the same hydrophobic interactions as kolanone and oruwacin (above). It is unlikely that these glycosides will remain intact *in vivo*, and hydrolysis may be necessary for absorption and general bioavailability [79]. Of the flavonoid aglycones from *M. morindoides*, apigenin, chrysoeriol, kaempferol, quercetin, and ombuin selectively docked with TbPTR1.

### Nauclea latifolia

Phytochemical investigations of *Nauclea latifolia* have revealed numerous indole alkaloids [40], [80], [81] (Table S13). The alkaloid glycosides 10-hydroxystrictosamide, cadambine, dihydrocadambine, and tetrahydrodesoxycordifoline showed notably strong docking to TbUDPGE, whereas non-glycosylated alkaloids showed preferential docking with TbPTR1and/or TbAK. 10-Hydroxyangustine, angustine, naucleamide B, and naucletine, in particular, docked more strongly with TbPTR1 than the co-crystallized ligand, 6-phenylpteridine-2,4,7-triamine [25] (docking energy = −27.6 kcal/mol).

### Newbouldia laevis

**Figure 9:**
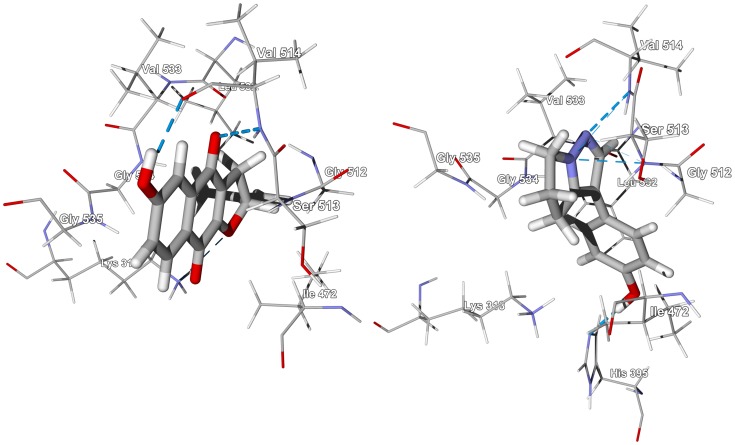
The crystal structure of *T. brucei* triosephosphate isomerase, TbTIM (PDB 1iih) [**50**]. The lowest-energy docking poses of 6-hydroxydehydroiso-α-lapachone (left) and 4*′*-hydroxywithasomnine (right) in the active site are shown. Hydrogen-bonding interactions are indicated by blue dashed lines.

*T. brucei* triosephosphate isomerase, TbTIM, is the likely protein target for the phytochemical agents of *Newbouldia laevis*. Both furanonaphthoquinones [82], [83] and pyrazole alkaloids [84], [85] from this plant showed remarkable selective affinity for this protein (Table S14). The monomeric furanonaphthoquinone ligands all occupy the same site with hydrogen bonding of the furan oxygen and C(9) carbonyl oxygen to Lys313; C(4) carbonyl oxygen with Ser513 and Val514; a van der Waals surface provided by Val533, Gly534, Gly535; and a hydrophobic pocket to accommodate the isopropenyl moiety provided by Ile472, Gly512, and Leu532 (see Fig. 9). Similarly, the pyrazole alkaloid 4*′*-hydroxywithasomnine has key hydrogen-bonding interactions between the pyrazole ring nitrogens and Ser513 and Val514. The aromatic ring lies in the hydrophobic pocket made up of Ile472 and Leu532, and there is an additional hydrogen-bonding interaction between the phenolic –OH group and His395 (Fig. 9).

### Physalis angulata

Withanolide triterpenoids, abundant components of *Physalis angulata* [40], [86]–[88], generally showed preferential docking to *T. brucei* sterol 14α-demethylase, TbCYP51 (Table S15). This is consistent with the docking of *L. inermis* triterpenoids and steroids, *C. procera* and *Khaya* limonoids (see above). Five of the withanolides, 14-hydroxyixocarpanolide, physagulin J, physagulin L, withangulatin H, and withangulatin I, docked more strongly to TbCYP51 than the co-crystallized ligand [16]. Three withanolides, physagulin A, physagulin L*′*, and withangulatin A, docked more strongly into TbODC than the co-crystallized ligand (pyridoxal 5*′*-phosphate) for that protein [34]. The pyrrolidine alkaloid, phygrine, docked with TbTIM preferentially.

### Picralima nitida

*Picralima nitida* glycosylated coumestans [89] showed strong binding to most of the protein targets, except for TbNDRT (Table S16). They are, for example, along with *Acacia nilotica* flavonoid gallates, the only ligands that dock to rhodesain with docking energies comparable to the co-crystallized ligand. Of the ligands examined in this work, coumestan **2** is the strongest-binding ligand for TbAK (−44.1 kcal/mol) and TbUDPGE (−43.7 kcal/mol). The corresponding aglycones, **4**, **5**, and **6**, however, were selective for TbPTR1 as well as TbUDPGE.

### Prosopis africana

The phytochemistry of *Prosopis africana* is characterized by piperidine alkaloids (Table S17) [40], [90]. These ligands exhibited similar docking energies with all protein targets, owing presumably to the small, flexible nature of the compounds. They did, however, show slightly better affinity for TbAK.

### Rauwolfia vomitoria

Phytochemical investigations of *Rauwolfia vomitoria* have revealed this plant to be replete with indole alkaloids (Table S18) [40], [91], [92]. The structural diversity of these indole alkaloids seems to defy targeting any one particular protein. There are some notable docking results, however. 3-Epirescinnamine docked with TbPRT1 and with TbODC more strongly than the co-crystallized ligands, 6-phenylpteridine-2,4,7-triamine [25] and putrescine [34], respectively. Isoreserpiline, raumitorine, and rauvanine also docked strongly to TbPTR1. Ajmalimine, isoreserpiline, rauvomitine, and serpenticine had remarkable docking energies with TbNH. The trimethoxybenzoyl and trimethoxycinnamyl esters, renoxydine, rescidine, rescinnamine, reserpine, along with methyl 3,4-dimethoxybenzoylreserpate, were all excellent ligands for TbUDPGE, with docking energies comparable to uridine-5*′*-diphosphate, the co-crystallized ligand [20]. Of these, renoxydine, rescidine, and reserpine, along with neonorreserpine, docked strongly to TbCYP51.

### Securidaca longipedunculata

**Figure 10:**
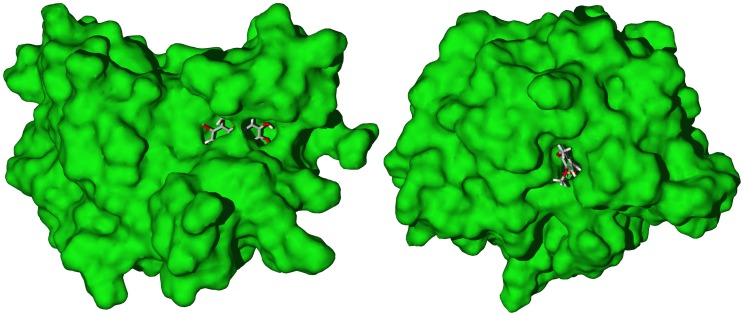
The crystal structure of *T. brucei* pteridine reductase 1 (TbPTR1, PDB 3jq7 [**43**]). Left: Lowest-energy docked pose of 1,3,6,8-tetrahydro-2,5-dimethoxyxanthone. Right: Lowest-energy docked pose of (*E*)-ethyl 4-methoxycinnamate with *T. brucei* triosephosphate isomerase (TbTIM, PDB 1iih [50]).

Cinnamate esters from *Securidaca longipedunculata* [40] showed selective docking to TbTIM while *S. longipedunculata* xanthones [40], [93] had a docking preference for TbPTR1. Both of these protein targets have relatively small binding sites, which are more suitable for the small ligands (Fig. 10). The xanthones also docked relatively strongly with TbUDPGE. The *S. longipedunculata* indole alkaloids dehydroelymoclavine and alkaloid A [94] docked strongly to TbPTR1 and TbAK, respectively, as well as with TbUDPGE (see Table S19).

### Strychnos spinosa

Phytochemicals isolated from *Strychnos spinosa* include secoiridoids [95], indole, pyridine, and naphthyridine alkaloids [40], sterols and triterpenoids [96] (Table S20). Relatively small pyridine and naphthyridine alkaloids from *Strychnos spinosa* showed preferential docking to TbPTR1 and/or TbTIM. The indole alkaloids akagerine, 10-hydroxyakagerine, and kribine also docked preferentially to TbPTR1, while iridoid glucosides preferred TbUDPGE. Both sterols and triterpenoids from *S. spinosa* selectively docked with TbCYP51, similar to what was observed with *L. inermis* sterols and triterpenoids (see above), but these compounds also showed notably strong docking with TbODC. Interestingly, a comparison of docking energies of triterpenoid and steroid ligands with their antitrypanosomal activities [96] shows no correlation, even comparing TbCYP51 docking or TbODC docking. Plots of log(*IC*_50_) vs. docking energies gives *R* ^2^ values of 0.043 and 0.007 for TbCYP51 and TbODC, respectively. It may be that inhibition of some other protein target [8], [9] is the biochemical mechanism of activity for these compounds.

In terms of natural products drug discovery, it is useful to examine whether different phytochemical classes show selectivity for particular protein targets. Simple flavonoid ligands showed docking preferences for TbPTR1 and TbUDPGE. Flavonoid gallates, on the other hand, were shown to be promiscuous docking ligands to all protein targets, but were particularly strongly docking with TbAK, TbPRT1, TbCYP51, and TbNH. Likewise, flavonoid glycosides tended to be promiscuous docking agents, but with preference for TbAK, TbPRT1, and TbNH. Oligomeric flavonoids (tannin-like polyphenolics) showed strong docking to TbAK. The diversity of flavonoid structures has led to diverse biological activities, including antiprotozoal activity, but the modes of antiprotozoal activity have not been well elucidated [97].

As previously noted (see above), triterpenoid ligands were largely selective for TbCYP51. Withanolide triterpenoids also showed a docking preference for TbCYP51, while limonoids preferentially docked with TbAK as well as TbCYP51. Not surprisingly, sterols showed a propensity to dock with TbCYP51, but also docked strongly with TbUDPGE.

All of the anthraquinone ligands examined in this docking study, docked with strong binding energies to TbPRT1. Likewise, xanthone ligands exhibited docking selectivity for TbPTR1. Naphthoquinones, on the other hand, docked preferentially with TbTIM. Most chromene ligands also showed notable docking energies to TbTIM. The phenylpropanoids examined showed preferences for TbTIM as well as TbUDPGE, while glycoside derivatives of phenylpropanoids showed selectivity for TbDHFR.

Berberine alkaloids docked preferentially to TbPTR1 while aporphine alkaloids showed some selectivity for TbPTR1 and TbUDPGE. Piperidine alkaloids were also selective for TbUDPGE. Pyrazole and pyridine alkaloids, on the other hand, preferred docking to TbTIM. A total of 93 indole alkaloids were examined in this docking study and many of them showed notable docking energies with TbUDPGE and some with TbAK and TbPTR1. Glycoside derivatives of alkaloids also preferentially docked with TbUDPGE.

Overall, the protein objects most targeted by the phytochemical ligands in this study were TbUDPGE, targeted by many alkaloids; TbPTR1, preferred by planar-like ligands; TbCYP51, which docked terpenoid ligands well; and TbAK, which docked many different classes of phytochemicals. Those proteins least preferred in terms of docking energies were rhodesain, TbCatB, and TbNDRT.

Rhodesain and TbCatB are both cysteine proteases with relatively small binding sites. It may be that the docking energies reflect the fact that only relatively small ligands, with inherently small docking energies, can fit well into the binding sites of these two proteins. The docking energies do not, however, reflect the potential for covalent bonding to the active sites of these proteins. It is useful, therefore, to examine small electrophilic ligands for energetically favorable docking orientations that would allow for reaction of nucleophilic amino acid side chains to the electrophilic sites of the ligands.

**Figure 11:**
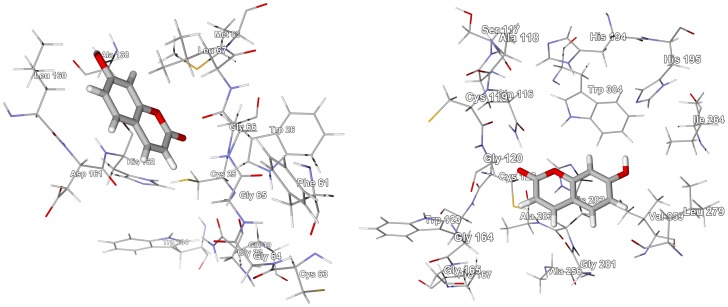
Docking poses of umbelliferone. Left: In the active sites of rhodesain (PDB 2p7u [40]). Right: In the active site of TbCatB (PDB 3hhi [46]).

Although umbelliferone does not dock with particularly strong energies to rhodesain or TbCatB, it does dock in poses such that the nucleophilic Cys25 of rhodesain or Cys122 of TbCatB are poised to undergo conjugate addition to the pyrone ring (Fig. 11). The S atom of Cys25 is 3.16 Å from C(4) of docked umbelliferone in rhodesain, while in TbCatB, Cys122 is 3.65 Å from C(4) of umbelliferone. Coumarins have been shown to be trypanocidal agents [98] and it has been suggested that umbelliferone undergoes conjugate addition with available cysteine thiol groups [99].

**Figure 12:**
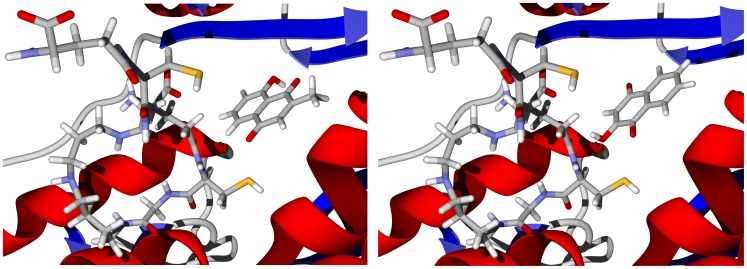
The crystal structure of *T. brucei* trypanothione reductase (TbTR, 2wow [**45**]). The docked poses are isoplumbagin (left) and lawsone (right) in the proximity of trypanothione.

**Figure 13:**
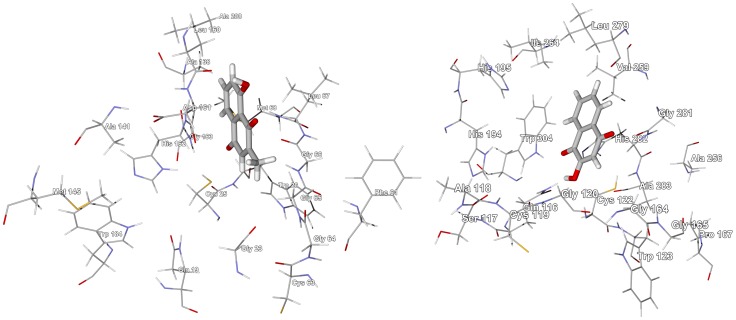
Left: Isoplumbagin in the active site of rhodesain (PDB 2p86 [41]. The S⋅⋅⋅C(3) = 3.18 Å. Right: Lowest-energy docked pose of lawsone in the active site of *T. brucei* cathepsin B (TbCatB, PDB 3hhi [46]; S⋅⋅⋅C(2) = 3.73 Å).

**Figure 14:**
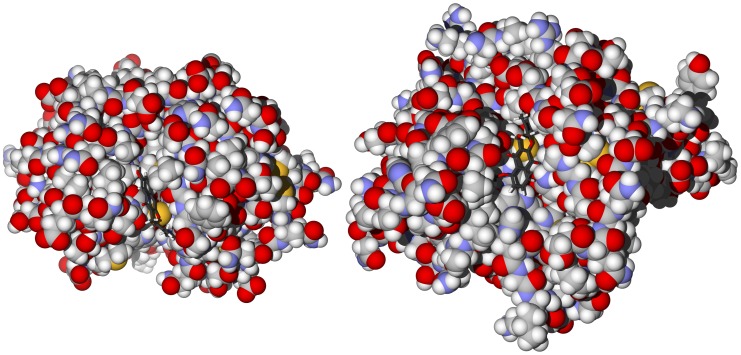
Lowest-energy docked poses of 6-hydroxydehydroiso-α-lapachone. Left: With rhodesain (PDB 2p86 [41]). Right: With TbCatB (PDB 3hhi [46]). Note the proximity and orientation of the quinone moiety with the cysteine sulfur atoms in the active sites.

Many naphthoquinones have been shown to be antitrypanosomal [100], and are suspected to interfere with redox thiol metabolism by inhibition of TbTR [101], [102]. There are docking poses, albeit not the lowest-energy poses, of isoplumbagin (docking pose energy = −9.9 kcal/mol) and lawsone (docking pose energy = −8.6 kcal/mol) with TbTR such that these quinone ligands are in the proximity of reduced trypanothione (Fig. 12). Similarly, both isoplumbagin and lawsone dock with the cysteine proteases rhodesain and TbCatB with the electrophilic carbons near the active-site cysteine residues (Fig. 13). *N. laevis* furanonaphthoquinones (α-lapachone derivatives) also dock with rhodesain in poses such that the nucleophilic Cys25 can undergo Michael addition to the quinone ring (Fig. 14). None of the furanonaphthoquinones docked near the trypanothione thiol groups in TbTR, however.

This *in-silico* investigation suggests that trypanosomal phytochemicals may target different protein targets. There are several caveats to these docking results: (a) many of the phytochemical agents may not be bioavailable due to limited solubility, membrane permeability, hydrolysis, or other metabolic decomposition; (b) tannins and other polyphenolics are promiscuous protein binding agents and are likely, therefore, not selective antitrypanosomal ligands; (c) the docking studies do not account for synergism in bioactivity of phytochemicals; (d) this current study does not address the binding of ligand to human homologous isozymes, which may also be targeted; (e) there are likely additional phytochemicals in each of the medicinal plants that have not been isolated or identified; and (f) there are likely additional trypanosomal proteins or other biochemical targets that have not yet been identified. Nevertheless, this *in-silico* molecular docking study has provided evidence for what phytochemical classes and structural manifolds are targeting particular trypanosomal protein targets and could provide the framework for synthetic modification of bioactive phytochemicals, *de novo* synthesis of structural motifs, and further phytochemical investigations.

## Supporting Information

Table S1 **Plants that have ethnopharmacological uses as antiprotozoal agents in West Africa.**
(DOCX)

Table S2 **Lowest-energy docking energies (kcal/mol) for** ***Acacia nilotica*** **phytochemicals with** ***Trypanosoma brucei*** **protein targets.**
(DOCX)

Table S3 **Lowest-energy docking energies (kcal/mol) for** ***Ageratum conyzoides*** **phytochemicals with** ***Trypanosoma brucei*** **protein targets.**
(DOCX)

Table S4 **Lowest-energy docking energies (kcal/mol) for** ***Annona senegalensis*** **phytochemicals with** ***Trypanosoma brucei*** **protein targets.**
(DOCX)

Table S5 **Lowest-energy docking energies (kcal/mol) for** ***Bridelia ferruginea*** **phytochemicals with** ***Trypanosoma brucei*** **protein targets.**
(DOCX)

Table S6 **Lowest-energy docking energies (kcal/mol) for** ***Carapa procera*** **phytochemicals with** ***Trypanosoma brucei*** **protein targets.**
(DOCX)

Table S7 **Lowest-energy docking energies (kcal/mol) for** ***Enantia chlorantha*** **phytochemicals with** ***Trypanosoma brucei*** **protein targets.**
(DOCX)

Table S8 **Lowest-energy docking energies (kcal/mol) for** ***Garcinia kola*** **phytochemicals with** ***Trypanosoma brucei*** **protein targets.**
(DOCX)

Table S9 **Lowest-energy docking energies (kcal/mol) for** ***Khaya ivorensis*** **and** ***Khaya senegalensis*** **phytochemicals with** ***Trypanosoma brucei*** **protein targets.**
(DOCX)

Table S10 **Lowest-energy docking energies (kcal/mol) for** ***Lawsonia inermis*** **phytochemicals with** ***Trypanosoma brucei*** **protein targets.**
(DOCX)

Table S11 **Lowest-energy docking energies (kcal/mol) for** ***Morinda lucida*** **phytochemicals with** ***Trypanosoma brucei*** **protein targets.**
(DOCX)

Table S12 **Lowest-energy docking energies (kcal/mol) for** ***Morinda morindoides*** **phytochemicals with** ***Trypanosoma brucei*** **protein targets.**
(DOCX)

Table S13 **Lowest-energy docking energies (kcal/mol) for** ***Nauclea latifolia*** **phytochemicals with** ***Trypanosoma brucei*** **protein targets.**
(DOCX)

Table S14 **Lowest-energy docking energies (kcal/mol) for** ***Newbouldia laevis*** **phytochemicals with** ***Trypanosoma brucei*** **protein targets.**
(DOCX)

Table S15 **Lowest-energy docking energies (kcal/mol) for** ***Physalis angulata*** **phytochemicals with** ***Trypanosoma brucei*** **protein targets.**
(DOCX)

Table S16 **Lowest-energy docking energies (kcal/mol) for** ***Picralima nitida*** **phytochemicals with** ***Trypanosoma brucei*** **protein targets.**
(DOCX)

Table S17 **Lowest-energy docking energies (kcal/mol) for** ***Prosopis africana*** **phytochemicals with** ***Trypanosoma brucei*** **protein targets.**
(DOCX)

Table S18 **Lowest-energy docking energies (kcal/mol) for** ***Rauwolfia vomitoria*** **phytochemicals with** ***Trypanosoma brucei*** **protein targets.**
(DOCX)

Table S19 **Lowest-energy docking energies (kcal/mol) for** ***Securidaca longipedunculata*** **phytochemicals with** ***Trypanosoma brucei*** **protein targets.**
(DOCX)

Table S20 **Lowest-energy docking energies (kcal/mol) for** ***Strychnos spinosa*** **phytochemicals with** ***Trypanosoma brucei*** **protein targets.**
(DOCX)
