# Supplementary material for: In-silico Investigation of Antitrypanosomal Phytochemicals from Nigerian Medicinal Plants
Source: PLoS Negl Trop Dis. 2012 Jul 24;6(7):e1727. doi: 10.1371/journal.pntd.0001727 (PMC3404109; doi:10.1371/journal.pntd.0001727)
Supplement: Table S1 — Plants that have ethnopharmacological uses as antiprotozoal agents in West Africa. (DOCX) [file pntd.0001727.s001.docx]

**Table S1.** Plants that have ethnopharmacological uses as antiprotozoal agents in West Africa.

| Plant | Traditional  Antiprotozoal Use | Antiprotozoal activity | Reference |
| --- | --- | --- | --- |
| *Acacia nilotica* (Fabaceae) | HAT | *T. b. rhodesiense* | [1] |
|  |  | *T. b. brucei* | [2] |
| *Acalypha hispida* (Euphorbiaceae) | HAT | *T. b. rhodesiense* | [1] |
| *Adenia lobata* (Passifloraceae) | HAT | *Trypanosoma brucei* | [3] |
| *Afzelia africana* (Fabaceae) | HAT |  | [4] |
| *Ageratum conyzoides* (Asteraceae) | HAT |  | [5,6] |
|  | HAT |  | [4] |
|  |  | *T. b. rhodesiense* | [7] |
| *Alchornea cordifolia* (Euphorbiaceae) | Malaria | *Trypanosoma brucei* | [3] |
| *Alstonia boonei* (Apocynaceae) | Malaria | *Trypanosoma brucei* | [3] |
|  | HAT | *T. b. rhodesiense* | [1] |
| *Annona senegalensis* (Annonaceae) | HAT | *T. b. rhodesiense* | [1] |
|  |  | *T. b. brucei* | [8] |
| *Anogeissus leiocarpus* (Combretaceae) | Malaria, HAT | *Trypanosoma brucei* | [3] |
| *Azadirachta indica* (Meliaceae) | Malaria | *Plasmodium falciparum* | [9] |
| *Balanites aegyptiacus* (Zygophyllaceae) | HAT |  | [4] |
| *Bobgunnia madagascarensis* (Fabaceae) | Malaria | *Leishmania major* | [10] |
| *Bridelia ferruginea* (Euphorbiaceae) | Malaria, HAT | *Trypanosoma brucei* | [3] |
|  |  | *Trypanosoma brucei* | [11] |
|  |  | *T. b. brucei* | [12] |
| *Bussea occidentalis* (Fabaceae) | HAT | *T. b. rhodesiense* | [1] |
| *Carapa procera* (Meliaceae) | HAT | *Trypanosoma brucei* | [3] |
| *Cassia sieberiana* (Fabaceae) | Malaria, HAT | *Leishmania major* | [10] |
| *Cissus quadrangularis* (Vitaceae) | Malaria |  | [10] |
|  |  | *T. b. brucei* | [2] |
| *Cordia myxa* (Boraginaceae) | HAT | *T. b. rhodesiense* | [1] |
| *Diospyros mespiliformis* (Ebenaceae) | HAT | *T. b. rhodesiense* | [1] |
| *Enantia chlorantha* (Annonaceae) | Malaria | *Plasmodium falciparum* | [13] |
| *Entada africana* (Fabaceae) | Malaria | *Leishmania major* | [10] |
|  |  | *T. b. rhodesiense* | [14] |
| *Garcinia kola* (Clusiaceae) | HAT |  | [15] |
| *Gardenia ternifolia* (Rubiaceae) | Malaria |  | [10] |
| *Glinus oppositifolius* (Aizoaceae) | Malaria | *Leishmania major* | [10] |
| *Haematostaphis barteri* (Anacardiaceae) | HAT |  | [5] |
| *Hymenocardia acida* (Euphorbiaceae) | HAT | *T. b. rhodesiense* | [1] |
| *Khaya grandifolia* (Meliaceae) | Malaria | *Plasmodium falciparum* | [9] |
|  |  |  | [13] |
| *Khaya ivorensis* (Meliaceae) | Malaria |  | [6] |
|  |  | *T.b.rhodesiense* | [14] |
| *Khaya senegalensis* (Meliaceae) | Malaria, Leishmaniasis, HAT |  | [10] |
|  | Malaria | *Plasmodium falciparum* | [9] |
|  |  | *Trypanosoma congolense* | [16] |
|  |  | *Trypanosoma brucei* | [11] |
|  | Trypanosomiasis |  | [17] |
| *Lawsonia inermis* (Lythraceae) | Malaria |  | [6] |
|  | HAT | *Trypanosoma brucei* | [3] |
|  |  | *Trypanosoma brucei* | [11] |
| *Lippia multiflora* (Verbenaceae) | Malaria | *Trypanosoma brucei* | [3] |
|  | HAT |  | [5] |
|  | HAT |  | [4] |
| *Mitragyna inermis* (Rubiaceae) | Malaria | *Leishmania major* | [10] |
| *Morinda lucida* (Rubiaceae) | Malaria | *Plasmodium falciparum* | [9] |
|  | Malaria, HAT | *Leishmania donovani* | [3] |
|  | HAT | *T. b. rhodesiense* | [1] |
|  |  | *T. b. brucei* | [18] |
| *Morinda morindoides* (Rubiaceae) |  | *T. b. rhodesiense* | [14] |
| *Nauclea latifolia* (Rubiaceae) | Malaria | *Plasmodium falciparum* | [9] |
|  |  | *Trypanosoma brucei* | [11] |
|  |  | *T. b. brucei* | [2] |
| *Newbouldia laevis* (Bignoniaceae) | Malaria |  | [6] |
|  | HAT | *T. b. rhodesiense* | [1] |
| *Opilia celtidifolia* (Opiliaceae) | Malaria | *Trypanosoma brucei* | [3] |
| *Paullinia pinnata* (Sabindaceae) | Malaria | *Trypanosoma brucei* | [3] |
| *Physalis angulata* (Solanaceae) | HAT | *T.b.rhodesiense* | [1] |
|  | HAT |  | [4] |
| *Picralima nitida* (Apocynaceae) | Malaria | *Plasmodium berghei* | [9] |
|  |  | *Plasmodium falciparum* | [13] |
| *Prosopis africana* (Fabaceae) | HAT | *Leishmania major* | [10] |
|  | Malaria | *Plasmodium falciparum* | [9] |
|  |  | *Trypanosoma brucei* | [11] |
|  | Trypanosomiasis |  | [17] |
| *Pseudocedrela kotschyi* (Meliaceae) | Malaria, HAT | *Leishmania major* | [10] |
| *Quassia amara* (Simaroubaceae) | Malaria | *Plasmodium falciparum* | [9] |
| *Rauwolfia vomitoria* (Apocynaceae) | Malaria |  | [15] |
| *Sarcocephalus latifolius* (Rubiaceae) | Malaria | *Leishmania major* | [10] |
| *Securidaca longipedunculata* (Polygalaceae) |  | *Trypanosoma congolense* | [16] |
|  | HAT |  | [4] |
| *Sida acuta* (Malvaceae) | Malaria | *Plasmodium falciparum* | [9] |
| *Spilanthes oleracea* (Oleraceae) | Malaria |  | [10] |
|  |  | *T. b. brucei* | [2] |
| *Strychnos spinosa* (Loganiaceae) | HAT | *T. b. rhodesiense* | [1] |
| *Tamarindus indica* (Fabaceae) | HAT |  | [10] |
| *Terminalia avicennioides* (Combretaceae) |  | *Trypanosoma brucei* | [16] |
|  |  | *T. b. brucei* | [2] |
| *Terminalia glaucescens* (Combretaceae) | Malaria | *Trypanosoma brucei* | [3] |
| *Tithonia diversifolia* (Asteraceae) | Malaria | *Plasmodium falciparum* | [9] |
| *Uvaria afzelii* (Annonaceae) | Malaria | *Leishmania donovani* | [3] |
| *Zanthoxylum zanthoxyloides* (Rutaceae) | Malaria, HAT | *Leishmania major* | [10] |
|  | HAT | *T. b. rhodesiense* | [1] |

**References for Table S1**

1. Freiburghaus F, Kaminsky R, Nkunya MHH, Brun R (1996) Evaluation of African medicinal plants for their in vitro trypanocidal activity. J Ethnopharmacol 55: 1-11.
2. Bizimana N, Tietjen U, Zessin KH, Diallo D, Djibril C, et al. (2006) Evaluation of medicinal plants from Mali for their in vitro and in vivo trypanocidal activity. J Ethnopharmacol 103: 350-356.
3. Okpekon T, Yolou S, Gleye C, Roblot F, Loiseau P, et al. (2004) Antiparasitic activities of medicinal plants used in Ivory Coast. J Ethnopharmacol 90: 91-97.
4. Ayensu ES (1978) Medicinal Plants of West Africa. Algonac, Michigan: Reference Publications.
5. Nigerian Medicinal Plants Database. Retrieved from http://www.scribd.com/doc/50835029/Nigerian-Plant-Database on 10/22/11.
6. Odugbemi T, Akinsulire O (2008) Medicinal plants species, family names and uses. In: A textbook of Medicinal Plants from Nigeria, Odugbemi T, ed. Lagos, Nigeria: University of Lagos Press. pp 541-612.
7. Nour AMM, Khalid SA, Kaiser M, Brun R, Abdalla WE, et al. (2010) The antiprotozoal activity of methylated flavonoids from *Ageratum conyzoides* L. J Ethnopharmacol 129: 127-130.
8. Ogbadoyi EO, Abdulganiy AO, Adama TZ, Okogun JI (2007) In vivo trypanocidal activity of *Annona senegalensis* Pers. leaf extract against *Trypanosoma brucei brucei*. J Ethnopharmacol 112: 85-89.
9. Abedayo JO, Krettli AU (2011) Potential antimalarials from Nigerian plants: A review. J Ethnopharmacol 133: 289-302.
10. Ahua KM, Ioset JR, Ioset KN, Diallo D, Mauël J, et al. (2007) Antileishmanial activities associated with plants used in the Malian traditional medicine. J Ethnopharmacol 110: 99-104.
11. Atawodi SE (2005) Comparative *in vitro* trypanocidal activities of petroleum ether, chloroform, methanol and aqueous extracts of some Nigerian savannah plants. Afr J Biotechnol 4: 177-182.
12. Ekanem JT, Kolawole OM, Abbah OC (2008) Trypanocidal potential of methanolic extract of *Bridelia ferruginea* benth bark in *Rattus novergicus*. Afr J Biochem Res 2: 45-50.
13. Kuete V, Efferth T (2010) Cameroonian medicinal plants: pharmacology and derived natural products. Front Pharmacol 1: 123.
14. Kamanzi Atindehou K, Schmid C, Brun R, Koné MW, et al. (2004) Antitrypanosomal and antiplasmodial activity of medicinal plants from Côte d’Ivoire. J Ethnopharmacol 90: 221-227.
15. Ndenecho EN (2009) Herbalism and resources for the development of Ethnopharmacology in Mount Cameroon region. Afr J Pharm Pharmacol 3: 78-86.
16. Atawodi SE, Bulus T, Ibrahim S, Ameh DA, Nok AJ, et al. (2003) *In vitro* trypanocidal effect of methanolic extract of some Nigerian savannah plants. Afr J Biotechnol 2: 317-321.
17. Maikai VA, Abubakar U, Salman AA, Inuwa TN (2010) Preliminary survey of medicinal plants used in treatment of animal trypanosomosis in Kaduna State, Nigeria. Ethnobot Leaf 14: 319-326.
18. Asuzu IU, Chineme CN (1990) Effects of *Morinda lucida* leaf extract on *Trypanosoma brucei brucei* infection in mice. J Ethnopharmacol 30: 307-313.
