# Supplementary material for: In-silico Investigation of Antitrypanosomal Phytochemicals from Nigerian Medicinal Plants
Source: PLoS Negl Trop Dis. 2012 Jul 24;6(7):e1727. doi: 10.1371/journal.pntd.0001727 (PMC3404109; doi:10.1371/journal.pntd.0001727)
Supplement: Table S2 — Lowest-energy docking energies (kcal/mol) for Acacia nilotica phytochemicals with Trypanosoma brucei protein targets. (DOCX) [file pntd.0001727.s002.docx]

**Table S2.** Lowest-energy docking energies (kcal/mol) for *Acacia nilotica* phytochemicals with *Trypanosoma brucei* protein targets.^a^

| Compound | Rhodesain | TbAK | TbPTR1 | TbDHFR | TbTR | TbCatB | TbHSP90 | TbCYP51 | TbNH | TbTIM | TbNDRT | TbUDPGE | TbODC |
| --- | --- | --- | --- | --- | --- | --- | --- | --- | --- | --- | --- | --- | --- |
|   1,3-Digalloylglucose | -27.5 | -35.5 | -34.7 | -33.9 | -33.2 | -28.9 | -31.2 | -33.6 | -35.5 | -31.0 | -34.6 | **-40.2** | -35.4 |
|   1,6-Digalloylglucose | -29.5 | -35.0 | -36.7 | -34.5 | -29.7 | -26.9 | -31.3 | -35.9 | **-37.2** | -33.4 | **-37.3** | -35.9 | -33.6 |
|   3*'*,5-Di*-O-*galloylcatechin | -33.3 | **-41.1** | -37.8 | -37.0 | -33.7 | -26.2 | -32.9 | **-38.4** | -38.0 | -28.0 | -31.3 | -36.6 | -35.2 |
|   3*'*,7-Di*-O-*galloylcatechin | -30.5 | -38.3 | -39.2 | -39.3 | -35.0 | -29.3 | -35.3 | -41.9 | **-44.2** | -29.0 | -41.7 | -37.1 | -40.4 |
|   3*'*,7-Di-*O*-galloylgallocatechin | -31.0 | -39.2 | -40.9 | -39.7 | -32.1 | -36.0 | -34.8 | -39.8 | **-41.2** | -30.3 | -34.4 | -37.4 | -38.0 |
|   3-*O*-Acetylhermaphrodiol | -17.8 | -20.7 | -23.0 | -20.4 | -19.2 | -18.2 | -19.5 | -20.1 | **-24.2** | -16.9 | -20.5 | **-24.2** | -23.0 |
|   4*'*,5-Di-*O*-galloylcatechin | -33.8 | -39.0 | -37.0 | -38.0 | -37.3 | -28.6 | -35.8 | **-41.1** | -38.3 | -29.7 | -34.8 | -38.0 | -38.8 |
|   4*'*,7-Di-*O*-galloylcatechin | -33.3 | -38.9 | -34.3 | -35.3 | -37.4 | -29.4 | -34.5 | **-40.5** | -35.4 | -26.4 | -40.1 | -39.0 | **-41.4** |
|   4*'*,7-Di-*O*-galloylgallocatechin | -32.9 | -39.5 | -33.9 | -37.1 | -35.0 | -29.7 | -34.4 | **-41.7** | -37.2 | -30.1 | -38.7 | -35.5 | -39.0 |
|   4*'*,7-Dimethoxynoranhydroicaritin-  3-*O-*β-d-xylopyranoside | -25.5 | -30.9 | -29.8 | -33.0 | -28.3 | -28.1 | -28.6 | -28.5 | **-35.1** | -30.1 | -10.2 | -28.6 | -30.0 |
|   5-*O-*Galloylcatechin | -25.2 | -36.9 | -33.9 | -33.9 | -26.9 | -27.4 | -29.8 | **-38.2** | -32.0 | -26.2 | -27.1 | -30.2 | -32.3 |
|   5-*O*-Galloylgallocatechin | -26.1 | **-37.3** | **-36.7** | -34.6 | -27.3 | -27.7 | -29.5 | **-38.5** | -31.9 | -28.9 | -29.2 | -31.3 | -32.0 |
|   5,7-Di-O-galloylcatechin | -32.9 | -37.8 | **-42.7** | -39.7 | -35.4 | -30.6 | -33.8 | -31.6 | **-41.1** | -30.8 | -32.5 | **-41.5** | -37.2 |
|   5,7-Di-*O*-galloylepigallocatechin | -30.8 | -38.7 | -36.0 | -38.9 | -33.5 | -29.4 | -33.4 | -34.9 | **-40.3** | -30.1 | -30.4 | -39.2 | -37.8 |
|   7-*O*-Galloylcatechin | -26.7 | -33.1 | **-35.3** | -32.4 | -27.5 | -26.4 | -27.9 | -29.7 | -31.7 | -29.0 | -32.2 | -33.5 | -30.7 |
|   7-*O*-Galloylepigallocatechin | -28.8 | **-34.3** | -33.6 | -33.2 | -29.0 | -26.3 | -29.4 | -28.4 | -32.9 | -29.0 | -31.8 | -33.9 | -29.6 |
|   7-*O*-Galloylgallocatechin | -27.7 | -33.6 | **-36.3** | -33.8 | -27.7 | -25.2 | -29.0 | -30.4 | -30.4 | -28.5 | -33.1 | -34.0 | -31.4 |
|   3,3*'*,4*'*,5,7-Pentahydroxy-2*'*,6,8-trimethoxy-3-*O*-  [α-l-rhamnopyranosyl-(1→2)-β-d-glucopyranoside] | -22.3 | **-36.2** | -29.1 | -28.6 | -29.9 | -21.4 | -31.3 | -25.3 | -31.8 | -26.6 | no dock | -10.8 | -31.3 |
|   Catechin | -19.9 | **-26.6** | -23.5 | -22.5 | -22.2 | -18.0 | -21.7 | -24.7 | -25.2 | -23.1 | -23.7 | -23.8 | -21.3 |
|   d-Pinitol | -14.0 | -16.4 | -16.1 | -17.2 | -17.4 | -12.5 | -17.8 | -16.1 | -16.9 | **-19.2** | -16.8 | -16.8 | -17.9 |
|   Methyl 4-galloylgallate | -23.6 | -27.5 | -28.6 | -29.8 | -27.4 | -22.5 | -28.2 | -26.2 | -28.8 | -30.4 | -29.9 | **-30.8** | -26.4 |
|   Methyl gallate | -15.4 | -18.0 | -19.3 | -18.5 | -18.2 | -15.2 | -17.8 | -16.6 | -16.7 | **-19.9** | -19.8 | -17.6 | -17.5 |
|   Niloticane | -14.4 | -22.7 | -23.0 | -17.7 | -21.1 | -20.0 | -18.5 | -21.8 | **-23.3** | -19.8 | -17.3 | -18.2 | -20.9 |
|   Umbelliferone | -14.6 | -15.9 | -17.6 | -14.7 | -16.1 | -12.7 | -16.0 | -14.6 | -15.1 | **-18.1** | -16.4 | -15.9 | -15.2 |

^a^Ligands showing selective (significantly stronger docking than average for all proteins) docking energies are highlighted in **blue bold**.
