# Supplementary material for: In-silico Investigation of Antitrypanosomal Phytochemicals from Nigerian Medicinal Plants
Source: PLoS Negl Trop Dis. 2012 Jul 24;6(7):e1727. doi: 10.1371/journal.pntd.0001727 (PMC3404109; doi:10.1371/journal.pntd.0001727)
Supplement: Table S3 — Lowest-energy docking energies (kcal/mol) for Ageratum conyzoides phytochemicals with Trypanosoma brucei protein targets. (DOCX) [file pntd.0001727.s003.docx]

**Table S3.** Lowest-energy docking energies (kcal/mol) for *Ageratum conyzoides* phytochemicals with *Trypanosoma brucei* protein targets.^a^

| Compound | Rhodesain | TbAK | TbPTR1 | TbDHFR | TbTR | TbCatB | TbHSP90 | TbCYP51 | TbNH | TbTIM | TbNDRT | TbUDPGE | TbODC |
| --- | --- | --- | --- | --- | --- | --- | --- | --- | --- | --- | --- | --- | --- |
|   3*'*,4*'*,5,5*'*,6,7-Hexamethoxyflavone | -22.6 | -27.7 | -25.2 | -23.9 | -24.7 | -18.2 | -20.8 | -21.6 | -26.9 | -25.8 | -19.9 | -27.7 | -23.7 |
|   3*'*,4*'*,5,5*'*,6,8-Hexamethoxyflavone | -23.3 | -28.0 | **-31.1** | -21.9 | -25.7 | -16.6 | -22.1 | -22.9 | -28.7 | -25.4 | -18.4 | **-30.3** | -24.1 |
|   3-Isobutyl-6,8-dimethoxy-2-methyl-4-chromone | -18.5 | -23.5 | -23.8 | -20.2 | -20.8 | -17.1 | -20.9 | -20.3 | -22.3 | -23.9 | -19.4 | -23.5 | -21.9 |
|   5,5*'*,6,7-Tetramethoxy-3*'*,4*'*-methylenedioxyflavone | -23.8 | -28.8 | **-30.7** | -24.2 | -25.8 | -18.8 | -24.9 | -25.0 | -29.6 | -25.1 | -21.9 | -28.5 | -26.3 |
|   5,6-Dimethoxy-2-isopropylbenzofuran | -17.9 | -20.0 | -20.8 | -17.2 | -20.2 | -15.7 | -20.8 | -21.5 | -20.5 | **-23.1** | -18.3 | -19.4 | -20.6 |
|   5*'*-Methoxynobiletine | -18.8 | -26.8 | **-30.6** | -23.8 | -25.8 | -17.9 | -21.9 | -23.0 | -28.0 | -22.0 | -11.9 | -29.4 | -21.0 |
|   6,7-Dimethoxy-2-methyl-2-(2-methyl-1-propanone)-3-chromene | -17.9 | -23.1 | -22.1 | -18.0 | -21.3 | -16.4 | -20.4 | -20.4 | -22.4 | **-23.3** | -20.0 | -22.0 | -19.9 |
|   6,7-Dimethoxy-2-methyl-2-isobutenyl-4-chromanone | -19.3 | **-24.1** | -22.3 | -19.3 | -21.5 | -16.3 | -20.4 | -20.9 | -22.0 | -21.9 | -19.4 | -22.2 | -20.6 |
|   6-Acetyl-2,2-dimethylchroman | -14.6 | -19.4 | -20.6 | -16.7 | -18.8 | -16.1 | -17.1 | -18.3 | -17.2 | **-21.9** | -15.8 | -17.0 | -16.6 |
|   6-Methoxy-2,2-dimethylchromene | -15.2 | -17.9 | -18.4 | -17.8 | -18.7 | -13.5 | -18.4 | -16.6 | -16.7 | -19.0 | -15.3 | -17.1 | -16.0 |
|   7-Methoxy-2,2-dimethylchromene | -16.3 | -17.8 | -18.4 | -16.9 | -17.9 | -13.0 | -18.2 | -18.4 | -17.1 | -19.2 | -16.2 | -17.6 | -16.8 |
|   8-Hydroxy-3*'*,4*'*,5,5',6,7-hexamethoxyflavone | -22.8 | -26.9 | -27.4 | -24.3 | -23.1 | -17.8 | -22.5 | -23.1 | -28.0 | -25.4 | -16.6 | **-28.7** | -24.7 |
|   Ageconylflavone C | -22.5 | **-28.9** | **-28.8** | -23.2 | -23.5 | -18.7 | -25.9 | -23.7 | -28.1 | -24.8 | -22.2 | -28.0 | -23.9 |
|   Ageratochromene | -15.9 | -18.9 | -19.7 | -16.2 | **-20.6** | -12.7 | -20.2 | -18.1 | -18.5 | -20.5 | -17.5 | -19.3 | -17.4 |
|   Ageratochromene dimer | -21.1 | -26.2 | **-28.2** | -25.8 | -25.4 | -22.3 | -23.6 | -26.6 | -25.2 | -27.3 | -18.0 | -24.5 | -26.5 |
|   Eupalestin | -22.0 | -27.7 | **-32.0** | -23.9 | -26.8 | -18.7 | -24.2 | -24.5 | -29.5 | -27.4 | -14.9 | -26.6 | -26.6 |
|   *O*-Methylencecalinol | -16.2 | -20.5 | -21.3 | -17.0 | -21.1 | -14.8 | -19.8 | -17.1 | -20.8 | **-21.6** | -18.7 | -19.4 | -18.8 |

^a^Ligands showing selective (significantly stronger docking than average for all proteins) docking energies are highlighted in **blue bold**.
