# Supplementary material for: In-silico Investigation of Antitrypanosomal Phytochemicals from Nigerian Medicinal Plants
Source: PLoS Negl Trop Dis. 2012 Jul 24;6(7):e1727. doi: 10.1371/journal.pntd.0001727 (PMC3404109; doi:10.1371/journal.pntd.0001727)
Supplement: Table S4 — Lowest-energy docking energies (kcal/mol) for Annona senegalensis phytochemicals with Trypanosoma brucei protein targets. (DOCX) [file pntd.0001727.s004.docx]

**Table S4.** Lowest-energy docking energies (kcal/mol) for *Annona senegalensis* phytochemicals with *Trypanosoma brucei* protein targets.^a^

| Compound | Rhodesain | TbAK | TbPTR1 | TbDHFR | TbTR | TbCatB | TbHSP90 | TbCYP51 | TbNH | TbTIM | TbNDRT | TbUDPGE | TbODC |
| --- | --- | --- | --- | --- | --- | --- | --- | --- | --- | --- | --- | --- | --- |
|   8,8*'*-Bisdihydrosiringenin | -25.9 | -28.9 | -29.3 | -30.1 | -27.4 | -21.4 | -28.4 | -26.6 | **-31.1** | -24.9 | -26.9 | -29.6 | -28.1 |
|   Annogalene | -27.2 | -34.0 | -31.4 | -30.6 | -28.4 | -29.6 | -32.7 | -33.3 | -35.2 | -25.7 | -28.3 | **-42.9** | -32.1 |
|   Annonacin | -26.8 | **-35.6** | -34.1 | -33.5 | -25.1 | -31.9 | -30.7 | -31.9 | -32.4 | -27.9 | -31.8 | **-37.2** | -33.1 |
|   Annonacin A | -28.7 | **-36.2** | -31.4 | -32.6 | -30.5 | -27.0 | -33.4 | **-36.4** | -33.6 | -25.2 | -31.4 | -32.4 | -33.8 |
|   Annosenegalin | -24.8 | **-35.8** | -33.3 | -28.5 | -28.4 | -26.6 | -28.2 | **-36.9** | -33.9 | -28.5 | -32.2 | -33.0 | -33.1 |
|   Elemol | -15.2 | -20.6 | -18.7 | -19.1 | -18.5 | -15.6 | -20.6 | -18.5 | -18.9 | -19.2 | -17.9 | -18.1 | -16.9 |
|   Isocorydine | -16.9 | **-23.6** | -18.2 | -19.9 | -20.7 | -14.8 | -21.8 | -20.1 | **-22.4** | -20.8 | -14.4 | -16.5 | -20.0 |
|   Methyl *ent-*16β-kauran-17,19-dioic acid | -14.2 | -18.0 | -18.4 | -19.4 | -21.0 | -19.1 | -14.4 | -22.2 | -18.2 | -10.4 | -17.5 | -20.7 | -20.9 |
|   Methyl *ent*-19-oxo-16b-kauran-17-oic acid | -16.2 | -18.9 | **-26.7** | -19.6 | -22.0 | -18.6 | -19.1 | -23.1 | -19.9 | -15.6 | -17.9 | -18.6 | -23.6 |
|   Roemerine | -18.0 | **-23.2** | -22.7 | -18.1 | -21.8 | -16.7 | -21.6 | -17.5 | -20.5 | -21.3 | -16.1 | -20.4 | -19.8 |
|   Senegalene | -30.3 | **-38.6** | -30.3 | -32.3 | -29.5 | -25.7 | -32.8 | **-38.2** | -36.2 | -27.5 | -31.7 | -26.8 | -34.2 |
|   β-Eudesmol | -10.4 | -18.1 | -18.0 | -16.1 | -19.3 | -15.9 | -21.5 | -17.4 | -18.4 | -18.3 | -17.8 | -18.3 | -17.1 |
|   *ent*-16β-Kauran-19-oic acid 16,17-diacetate | -17.3 | -22.6 | -19.3 | -22.5 | -24.1 | -19.0 | -22.4 | **-28.1** | -23.0 | -24.9 | -18.7 | -26.5 | -21.6 |
|   *ent*-19-Oxo-16β-kauran-17-oic acid | -15.7 | -18.0 | **-24.0** | -18.9 | -20.3 | -19.2 | -16.6 | -22.1 | -18.2 | -11.4 | -16.7 | -17.7 | -21.6 |
|   γ-Eudesmol | -14.7 | -19.7 | -19.2 | -15.9 | -18.8 | -15.6 | -21.7 | -16.2 | -18.9 | -18.6 | -16.2 | -18.7 | -18.1 |

^a^Ligands showing selective (significantly stronger docking than average for all proteins) docking energies are highlighted in **blue bold**.
