# Supplementary material for: In-silico Investigation of Antitrypanosomal Phytochemicals from Nigerian Medicinal Plants
Source: PLoS Negl Trop Dis. 2012 Jul 24;6(7):e1727. doi: 10.1371/journal.pntd.0001727 (PMC3404109; doi:10.1371/journal.pntd.0001727)
Supplement: Table S5 — Lowest-energy docking energies (kcal/mol) for Bridelia ferruginea phytochemicals with Trypanosoma brucei protein targets. (DOCX) [file pntd.0001727.s005.docx]

**Table S5.** Lowest-energy docking energies (kcal/mol) for *Bridelia ferruginea* phytochemicals with *Trypanosoma brucei* protein targets.^a^

| Compound | Rhodesain | TbAK | TbPTR1 | TbDHFR | TbTR | TbCatB | TbHSP90 | TbCYP51 | TbNH | TbTIM | TbNDRT | TbUDPGE | TbODC |
| --- | --- | --- | --- | --- | --- | --- | --- | --- | --- | --- | --- | --- | --- |
|   5*'*-Demethoxy-β-peltatin A glucoside | -26.4 | **-36.3** | -28.6 | -32.2 | -29.7 | -28.6 | -31.9 | -30.9 | -29.6 | -26.8 | -17.1 | -32.5 | -33.4 |
|   Delphinidin | -22.9 | -25.0 | **-26.1** | -21.9 | -23.6 | -18.6 | -23.5 | -21.3 | -25.1 | -24.7 | -24.3 | -25.7 | -23.3 |
|   Epigallocatechin(7→4*'*)gallocatechin | -22.5 | -32.5 | -30.7 | -26.7 | -31.5 | -30.3 | -29.1 | **-39.1** | -28.1 | -25.5 | -33.3 | -33.3 | -33.9 |
|   Ferrugin | -24.8 | -27.5 | **-30.0** | -23.2 | -26.0 | -17.7 | -25.6 | -23.7 | -26.3 | -25.1 | -24.1 | **-30.0** | -24.9 |
|   Friedelin | no dock | -20.0 | -16.3 | -18.0 | -18.6 | -16.3 | -16.1 | -24.5 | -5.0 | -18.7 | -8.5 | **-23.6** | -17.7 |
|   Taraxerol | -9.7 | -20.5 | -16.0 | -19.5 | -18.5 | -14.8 | -20.2 | -24.2 | -18.3 | -12.6 | -13.2 | **-25.1** | -22.9 |

^a^Ligands showing selective (significantly stronger docking than average for all proteins) docking energies are highlighted in **blue bold**.
