# Supplementary material for: In-silico Investigation of Antitrypanosomal Phytochemicals from Nigerian Medicinal Plants
Source: PLoS Negl Trop Dis. 2012 Jul 24;6(7):e1727. doi: 10.1371/journal.pntd.0001727 (PMC3404109; doi:10.1371/journal.pntd.0001727)
Supplement: Table S6 — Lowest-energy docking energies (kcal/mol) for Carapa procera phytochemicals with Trypanosoma brucei protein targets. (DOCX) [file pntd.0001727.s006.docx]

**Table S6.** Lowest-energy docking energies (kcal/mol) for *Carapa procera* phytochemicals with *Trypanosoma brucei* protein targets.^a^

| Compound | Rhodesain | TbAK | TbPTR1 | TbDHFR | TbTR | TbCatB | TbHSP90 | TbCYP51 | TbNH | TbTIM | TbNDRT | TbUDPGE | TbODC |
| --- | --- | --- | --- | --- | --- | --- | --- | --- | --- | --- | --- | --- | --- |
|   3β-Isobutyryloxy-1-oxomeliac-8(30)-enate | -20.8 | **-27.0** | **-27.8** | -20.7 | -19.3 | -24.3 | -22.2 | -24.6 | -21.0 | -23.3 | -18.2 | -20.1 | **-27.1** |
|   6-Deoxyswietenolide | -15.7 | -23.2 | -17.6 | -23.7 | -20.0 | -20.6 | -22.9 | -20.9 | -0.6 | -11.8 | -14.8 | -24.0 | -23.0 |
|   *Carapa* spirolactone | -17.4 | -22.6 | -18.9 | -20.1 | -22.6 | -19.6 | -20.6 | **-26.1** | -17.7 | -18.4 | -13.8 | -23.6 | -21.4 |
|   Carapin | -15.0 | -24.3 | -19.3 | -24.4 | -22.8 | -12.9 | -23.5 | **-27.8** | -27.1 | -22.8 | -13.8 | -25.1 | **-27.6** |
|   Carapolide A | -12.8 | -27.4 | -24.9 | -24.7 | -24.0 | -23.9 | -25.7 | **-31.8** | -26.9 | -22.6 | -17.9 | -27.7 | -27.6 |
|   Carapolide B | no dock | -25.4 | -21.2 | -20.8 | -21.8 | -22.4 | -22.0 | **-29.3** | -15.9 | -13.9 | -20.0 | -27.6 | -25.4 |
|   Carapolide C | -21.4 | -24.2 | -24.3 | -23.3 | -23.8 | -25.9 | -19.0 | **-28.5** | -4.7 | -5.1 | -8.7 | -24.7 | -25.7 |
|   Evodulone | -18.1 | -22.9 | -19.3 | -24.2 | -22.3 | **-26.1** | -19.1 | -25.2 | -16.9 | -24.0 | -16.4 | -26.2 | -24.7 |
|   Mexicanolide | -21.1 | **-24.5** | -18.6 | -22.9 | -20.5 | -20.9 | -19.8 | -22.9 | -13.0 | -12.1 | -0.4 | -23.3 | -21.1 |
|   Proceranone | -15.5 | -23.3 | -18.9 | -23.8 | -22.6 | -22.6 | -19.8 | -25.8 | -18.5 | -22.2 | -15.3 | -27.7 | **-26.0** |
|   Procerin | -8.3 | **-25.5** | -19.4 | -15.9 | -21.4 | -22.5 | -13.7 | **-27.5** | -13.0 | -5.6 | -7.3 | -13.6 | -19.5 |

^a^Ligands showing selective (significantly stronger docking than average for all proteins) docking energies are highlighted in **blue bold**.
