# Supplementary material for: In-silico Investigation of Antitrypanosomal Phytochemicals from Nigerian Medicinal Plants
Source: PLoS Negl Trop Dis. 2012 Jul 24;6(7):e1727. doi: 10.1371/journal.pntd.0001727 (PMC3404109; doi:10.1371/journal.pntd.0001727)
Supplement: Table S7 — Lowest-energy docking energies (kcal/mol) for Enantia chlorantha phytochemicals with Trypanosoma brucei protein targets. (DOCX) [file pntd.0001727.s007.docx]

**Table S7.** Lowest-energy docking energies (kcal/mol) for *Enantia chlorantha* phytochemicals with *Trypanosoma brucei* protein targets.^a^

| Compound | Rhodesain | TbAK | TbPTR1 | TbDHFR | TbTR | TbCatB | TbHSP90 | TbCYP51 | TbNH | TbTIM | TbNDRT | TbUDPGE | TbODC |
| --- | --- | --- | --- | --- | --- | --- | --- | --- | --- | --- | --- | --- | --- |
|   (*S*)-Isoboldine | -18.9 | -23.8 | -25.0 | -21.6 | -22.1 | -19.4 | -23.9 | -22.3 | -23.7 | -21.6 | -15.1 | **-27.1** | -22.5 |
|   (*S*)-Isocorydine | -18.6 | **-24.3** | -23.9 | -20.9 | -22.9 | -17.3 | -24.1 | -20.2 | -22.5 | -20.2 | -13.5 | **-24.5** | -21.6 |
|   6*a*,7-Didehydro-7-hydroxy-12-dimethoxyaporphine | -16.1 | -22.4 | -20.2 | -17.9 | -21.9 | -16.3 | **-23.7** | -16.1 | -21.1 | -21.1 | -13.4 | -22.4 | -20.4 |
|   6*a*,7-Didehydro-7-hydroxy-12-dimethoxynoraporphine | -16.1 | -22.8 | **-25.8** | -19.7 | -22.4 | -16.4 | -24.4 | -18.0 | -21.1 | -22.2 | -14.5 | -21.3 | -20.6 |
|   8-Hydroxypalmatine | -21.5 | -24.2 | **-26.4** | -19.4 | -21.1 | -16.6 | -21.7 | -24.0 | -23.9 | -24.9 | -20.0 | **-26.7** | -22.2 |
|   *O*-Methylmoschatoline | -19.2 | -23.2 | -22.6 | -18.0 | -23.2 | -14.3 | -22.5 | -18.8 | -21.4 | -21.4 | -11.9 | -22.8 | -21.1 |
|   Argentinine | -20.1 | -24.1 | **-26.1** | -22.4 | -23.9 | -17.3 | -24.7 | -22.4 | -23.3 | -24.1 | -17.7 | -25.6 | -21.1 |
|   Atherosperminine | -18.7 | -23.9 | **-26.2** | -20.8 | -23.6 | -16.6 | -21.9 | -20.6 | -23.7 | -24.9 | -16.7 | -25.5 | -22.2 |
|   Columbamine | -21.6 | **-24.6** | -24.2 | -19.4 | -22.1 | -17.4 | -22.8 | -22.1 | -22.9 | **-24.7** | -18.0 | -24.0 | -21.5 |
|   Jatrorrhizine | -18.1 | -24.9 | **-25.8** | -21.3 | -22.4 | -15.7 | -23.8 | -21.8 | -23.6 | -22.4 | -20.0 | -25.2 | -23.1 |
|   Lanuginosine | -19.4 | -24.6 | **-26.1** | -19.9 | -24.1 | -16.6 | -22.5 | -20.9 | -23.1 | -24.0 | -15.1 | **-26.2** | -21.8 |
|   Liriodenine | -19.1 | -22.3 | -23.9 | -19.2 | -22.6 | -18.2 | -23.2 | -18.7 | -21.4 | **-24.0** | -14.1 | -23.8 | -20.5 |
|   Lysicamine | -18.7 | **-23.7** | -23.9 | -18.1 | -22.2 | -16.1 | **-24.1** | -18.6 | -21.0 | -20.1 | -14.3 | -23.5 | -21.0 |
|   Palmatine | -19.8 | -24.3 | -24.7 | -20.5 | -24.4 | -14.2 | -23.3 | -23.2 | -22.9 | -23.2 | -20.5 | -23.2 | -21.1 |
|   Pseudocolumbamine | -21.6 | -25.3 | **-27.5** | -20.2 | -22.8 | -18.3 | -23.4 | -22.2 | -25.0 | -24.9 | -20.4 | -26.0 | -23.5 |
|   Pseudopalmatine | -19.3 | -25.0 | **-27.5** | -20.9 | -23.5 | -17.9 | -23.4 | -20.9 | -24.6 | -23.0 | -20.2 | -25.8 | -21.6 |

^a^Ligands showing selective (significantly stronger docking than average for all proteins) docking energies are highlighted in **blue bold**.
