# Supplementary material for: In-silico Investigation of Antitrypanosomal Phytochemicals from Nigerian Medicinal Plants
Source: PLoS Negl Trop Dis. 2012 Jul 24;6(7):e1727. doi: 10.1371/journal.pntd.0001727 (PMC3404109; doi:10.1371/journal.pntd.0001727)
Supplement: Table S8 — Lowest-energy docking energies (kcal/mol) for Garcinia kola phytochemicals with Trypanosoma brucei protein targets. (DOCX) [file pntd.0001727.s008.docx]

**Table S8.** Lowest-energy docking energies (kcal/mol) for *Garcinia kola* phytochemicals with *Trypanosoma brucei* protein targets.^a^

| Compound | Rhodesain | TbAK | TbPTR1 | TbDHFR | TbTR | TbCatB | TbHSP90 | TbCYP51 | TbNH | TbTIM | TbNDRT | TbUDPGE | TbODC |
| --- | --- | --- | --- | --- | --- | --- | --- | --- | --- | --- | --- | --- | --- |
|   1,2,8-Trimethoxyxanthone | -17.8 | -20.5 | **-23.9** | -17.6 | -19.4 | -14.7 | -19.9 | -18.1 | -20.3 | **-22.6** | -18.1 | -22.2 | -19.5 |
|   4',5,7-Trimethoxyflavone | -19.6 | -25.7 | **-27.7** | -20.8 | -22.7 | -21.8 | -21.3 | -23.1 | -24.8 | -23.3 | -19.7 | -22.2 | -23.2 |
|   GB 1 | -21.3 | **-32.4** | -25.2 | -26.5 | -25.8 | -22.5 | -27.9 | -26.5 | -22.5 | -25.3 | -17.9 | -16.7 | -28.7 |
|   GB 1a | -20.0 | **-31.4** | -24.2 | -24.3 | -23.6 | -22.9 | -26.8 | -26.4 | -21.1 | -23.8 | -19.9 | -22.0 | **-29.8** |
|   GB 2 | -22.8 | **-32.7** | -7.9 | -24.7 | -24.3 | -23.5 | -25.1 | -26.9 | -23.5 | -27.0 | -20.4 | -19.8 | -29.3 |
|   GB 2a | -24.8 | **-30.3** | -23.3 | -25.6 | -26.6 | -24.6 | **-31.8** | -27.9 | -26.0 | -22.3 | -18.7 | -27.4 | -28.3 |
|   GB 2b | -23.2 | **-30.5** | -21.6 | -27.7 | -27.5 | -24.5 | **-31.4** | -25.0 | -21.5 | -25.2 | -17.1 | -23.8 | -28.1 |
|   GB 3 | -22.3 | **-31.2** | -30.9 | -26.9 | -24.4 | -26.0 | -25.8 | -30.3 | -23.9 | -26.1 | -14.9 | -26.9 | **-31.5** |
|   Garcifuran B | -20.7 | -23.6 | **-24.5** | -23.3 | -21.7 | -19.4 | -22.8 | -21.5 | -23.0 | -23.8 | -22.7 | -21.9 | -22.3 |
|   Garcinal | -27.3 | -31.7 | **-32.9** | -28.0 | -27.5 | -26.7 | -31.4 | -30.9 | -31.1 | -24.7 | -24.9 | **-34.5** | -30.1 |
|   Garcinianin | -24.6 | -28.9 | -23.6 | -27.4 | -28.2 | -26.1 | -27.1 | -24.7 | **-32.7** | -20.7 | -20.9 | -22.4 | -28.1 |
|   Garciniflavanone | -23.0 | **-33.7** | -19.3 | -24.0 | -23.3 | -24.3 | -24.6 | -29.1 | -23.5 | -27.1 | -18.0 | -20.3 | **-30.0** |
|   Garcinoic acid | -27.4 | -33.2 | -31.2 | -30.4 | -27.8 | -25.2 | -28.8 | -30.9 | -32.9 | -24.3 | -29.2 | **-35.9** | -29.2 |
|   Garcinol | -22.7 | **-26.3** | -26.0 | -23.4 | -23.3 | -20.6 | -24.7 | -21.2 | -25.1 | -24.8 | -21.4 | -24.0 | -23.3 |
|   Garcipyran | -19.8 | -26.0 | -25.0 | -20.8 | -24.7 | -18.0 | -24.2 | -26.3 | -25.1 | **-27.4** | -24.2 | -23.1 | -24.2 |
|   Kolaflavanone | -19.8 | **-30.8** | -20.1 | -26.1 | -26.4 | -22.8 | -26.4 | -27.0 | -24.3 | -29.0 | -17.4 | -12.8 | **-30.3** |
|   Kolanone | -28.4 | **-35.6** | -34.3 | -30.1 | -28.3 | -24.8 | -29.0 | -29.7 | **-37.1** | -32.3 | -28.6 | -33.8 | -32.6 |

^a^Ligands showing selective (significantly stronger docking than average for all proteins) docking energies are highlighted in **blue bold**.
