# Supplementary material for: In-silico Investigation of Antitrypanosomal Phytochemicals from Nigerian Medicinal Plants
Source: PLoS Negl Trop Dis. 2012 Jul 24;6(7):e1727. doi: 10.1371/journal.pntd.0001727 (PMC3404109; doi:10.1371/journal.pntd.0001727)
Supplement: Table S9 — Lowest-energy docking energies (kcal/mol) for Khaya ivorensis and Khaya senegalensis phytochemicals with Trypanosoma brucei protein targets. (DOCX) [file pntd.0001727.s009.docx]

**Table S9.** Lowest-energy docking energies (kcal/mol) for *Khaya ivorensis* and *Khaya senegalensis* phytochemicals with *Trypanosoma brucei* protein targets.^a^

| Compound | Rhodesain | TbAK | TbPTR1 | TbDHFR | TbTR | TbCatB | TbHSP90 | TbCYP51 | TbNH | TbTIM | TbNDRT | TbUDPGE | TbODC |
| --- | --- | --- | --- | --- | --- | --- | --- | --- | --- | --- | --- | --- | --- |
|   1-*O*-Acetylkhayanolide A | -16.0 | **-26.9** | -11.7 | -24.4 | -23.1 | -21.7 | -21.2 | -23.7 | -9.2 | -7.7 | no dock | -22.0 | -25.1 |
|   1-*O*-Acetylkhayanolide B | -15.3 | **-27.3** | -15.4 | -22.3 | -21.9 | -22.0 | -19.0 | -23.1 | -9.0 | -12.6 | no dock | -24.4 | -25.7 |
|   1-*O*-Deacetyl-2α-hydroxykhayanolide E | -11.4 | -25.3 | -15.7 | -15.8 | -24.9 | -25.2 | -20.9 | **-28.9** | -0.4 | no dock | no dock | -24.8 | -24.0 |
|   1-Deacetyl-6-deoxykhayanolide E | -14.1 | -24.8 | -17.0 | -21.2 | -22.6 | -25.4 | -21.2 | -26.9 | no dock | -3.8 | -6.6 | -23.6 | -22.2 |
|   1-Deacetylkhivorin | -5.4 | -23.8 | -18.1 | -21.7 | -21.9 | -20.8 | -22.0 | -21.8 | no dock | -9.1 | no dock | -14.2 | -24.7 |
|   11α-Acetoxy-2α-hydroxy-6-deoxyswietenine acetate | -16.9 | **-28.8** | -20.7 | -24.5 | -20.4 | -18.4 | -23.1 | -21.4 | -20.6 | -16.0 | no dock | -24.1 | -23.5 |
|   1,3,7-Trideacetylkhivorin | -12.4 | -21.6 | -15.1 | -10.5 | -19.4 | **-24.2** | -20.7 | -21.9 | -13.1 | -20.1 | -8.4 | **-26.3** | -23.5 |
|   3-*O*-Acetylanthothecanolide | -23.3 | **-27.0** | -16.1 | -25.3 | -23.0 | -26.8 | -18.2 | -22.6 | -21.7 | -16.8 | -14.2 | -25.9 | **-27.5** |
|   3-*O*-Acetylswietenine | -13.4 | **-27.6** | -24.2 | -18.2 | -20.1 | -21.1 | -20.4 | -21.7 | -1.5 | -20.3 | no dock | -21.3 | -25.3 |
|   3-*O*-Acetylswietenolide | no dock | **-25.6** | -19.3 | -23.9 | -20.0 | -21.3 | -20.0 | -21.3 | no dock | -14.4 | no dock | -22.9 | -24.8 |
|   3-*O*-Acetylkhayalactone | -21.3 | **-31.6** | -22.3 | **-32.2** | -24.6 | -24.4 | -22.0 | -30.6 | -19.5 | no dock | no dock | **-34.2** | -26.8 |
|   3-Deacetylkhivorin | -4.1 | **-25.7** | -20.0 | -18.1 | -20.5 | -21.1 | -22.1 | -20.4 | no dock | -16.2 | no dock | -22.3 | -23.4 |
|   3,7-Dideacetylkhivorin | -4.1 | -24.5 | -18.4 | -17.6 | -19.3 | **-27.2** | -21.5 | -19.9 | -10.6 | -18.5 | -6.6 | **-27.7** | -24.9 |
|   6-*O*-Acetylswietenolide | -14.8 | -24.5 | -21.6 | -25.1 | -19.9 | -24.0 | -21.5 | -21.9 | -5.9 | -12.4 | no dock | -23.7 | -23.8 |
|   6-Hydroxykhayalactone | -19.9 | -27.1 | -21.2 | -25.8 | -25.0 | -23.0 | -21.3 | **-29.9** | -18.6 | no dock | -2.4 | -28.7 | -26.7 |
|   7-Deacetyl-7-oxogedunin | -15.8 | -21.9 | -19.0 | -21.8 | **-24.0** | -18.3 | -18.2 | -23.0 | **-23.6** | -19.7 | -19.5 | -22.3 | -23.0 |
|   7-Deacetylgedunin | -12.9 | -23.3 | -18.5 | -18.2 | -23.3 | -22.0 | -18.8 | -22.8 | -21.7 | -4.4 | -14.8 | **-26.2** | -24.6 |
|   7-Deacetylkhivorin | -6.5 | -25.8 | -14.8 | -22.3 | -19.0 | -27.9 | -24.4 | -24.7 | no dock | -21.0 | -3.3 | -27.3 | -25.3 |
|   Anthothecanolide | -16.9 | -25.3 | -20.2 | -23.0 | -25.0 | -23.7 | -20.1 | **-29.7** | -19.5 | -16.9 | no dock | -25.9 | -24.0 |
|   Deacetylkhayanolide E | -14.6 | -24.4 | -16.1 | -20.4 | -23.8 | -25.8 | -18.3 | **-29.7** | no dock | no dock | -5.7 | -23.4 | -23.1 |
|   Fissinolide | -6.6 | **-26.0** | -17.2 | **-27.0** | -19.9 | -20.7 | -19.7 | -21.4 | -1.8 | -13.0 | no dock | -24.2 | -23.2 |
|   Gedunin | -4.3 | -25.4 | -18.6 | -19.9 | -21.6 | -25.6 | -20.4 | -22.2 | -2.2 | -15.4 | -4.3 | -21.8 | -22.9 |
|   Grandifolide A | no dock | **-26.8** | -21.1 | **-26.9** | -21.6 | -21.3 | -22.7 | -22.9 | -20.7 | -16.8 | no dock | -24.7 | **-27.4** |
|   Grandifolin | -11.8 | -25.9 | -10.5 | -24.6 | -24.4 | -18.8 | -23.0 | -24.7 | no dock | -4.1 | no dock | -20.6 | -26.9 |
|   Grandifoliolenone | -10.3 | -21.8 | -16.3 | -23.2 | -22.1 | -21.8 | -18.2 | **-30.8** | -14.8 | -17.6 | -12.5 | **-27.2** | -21.7 |
|   Grandifotane | -10.7 | **-29.3** | -20.7 | -26.0 | -22.0 | -24.3 | -21.9 | -25.1 | no dock | -6.7 | no dock | **-29.9** | -26.3 |
|   Khayalactone | -20.1 | -28.0 | -18.2 | -28.3 | -23.3 | -23.2 | -21.6 | **-29.8** | -19.8 | -17.6 | no dock | **-32.8** | -27.4 |
|   Khayanolide A | -16.7 | -25.3 | -2.7 | -24.9 | -19.7 | -22.2 | -23.3 | **-30.3** | -18.5 | -10.1 | no dock | -23.4 | -22.9 |
|   Khayanolide B | -16.1 | -25.5 | -12.0 | -19.6 | -22.3 | -26.1 | -19.8 | **-28.0** | no dock | no dock | -5.6 | -25.1 | -24.1 |
|   Khayanoside | -18.4 | **-32.1** | -22.7 | -23.1 | -28.2 | -23.9 | -22.4 | -28.8 | -19.7 | no dock | -1.9 | -26.1 | -28.8 |
|   Khivorin | -17.3 | -27.2 | -5.7 | -24.8 | -21.9 | -26.6 | -23.6 | -22.6 | -9.6 | -12.3 | no dock | -23.0 | -26.6 |
|   Methyl 6-acetoxyangolensate | -12.0 | -23.3 | -13.4 | -24.5 | -21.3 | -23.2 | -16.2 | **-25.1** | -15.1 | -15.5 | no dock | -17.2 | -24.1 |
|   Methyl angolensate | -18.1 | -22.5 | -22.1 | -22.8 | -18.9 | -21.8 | -16.0 | **-25.9** | no dock | -14.8 | no dock | -15.8 | -21.0 |
|   Methyl 6-hydroxyangolensate | -16.7 | -23.4 | -21.2 | -22.3 | -19.5 | -21.4 | -18.8 | **-25.8** | -10.1 | -6.4 | no dock | -20.9 | -22.6 |
|   Methyl ivorensate | -15.9 | -22.2 | -19.9 | -24.1 | -22.1 | -22.9 | -18.3 | -22.5 | -8.2 | -16.2 | -6.2 | -21.1 | **-25.6** |
|   Mexicanolide | -21.1 | **-24.5** | -18.6 | -22.9 | -20.5 | -20.9 | -19.8 | -22.9 | -13.0 | -12.1 | -0.4 | -23.3 | -21.1 |
|   Proceranolide | -9.4 | -23.8 | -19.3 | -23.2 | -20.1 | -21.4 | -19.9 | -20.7 | no dock | -11.1 | -11.4 | -25.1 | -23.0 |
|   Proceranolide butanoate | -14.0 | **-27.9** | -18.9 | **-28.3** | -20.8 | -20.6 | -24.0 | -21.9 | -16.9 | -14.8 | no dock | -25.1 | -25.2 |
|   Seneganolide | -19.1 | -26.1 | -23.5 | -19.7 | -21.9 | -20.8 | -19.0 | **-29.9** | -16.2 | -12.8 | no dock | -25.6 | -22.0 |
|   Swiemahogin A | -18.1 | -25.4 | -24.9 | **-28.1** | -25.1 | -21.4 | -23.1 | -26.7 | -23.6 | no dock | -22.0 | **-28.5** | -26.1 |
|   Swietenine | -12.3 | **-30.0** | -24.9 | -16.7 | -23.6 | -25.5 | -25.0 | -26.5 | -13.2 | -17.1 | no dock | -24.3 | **-28.9** |
|   Swietenolide | -8.4 | -23.8 | -18.0 | -21.7 | -20.5 | -21.4 | -19.8 | -22.2 | -6.1 | -13.4 | -13.5 | -23.8 | -22.5 |

^a^Ligands showing selective (significantly stronger docking than average for all proteins) docking energies are highlighted in **blue bold**.
