# Supplementary material for: In-silico Investigation of Antitrypanosomal Phytochemicals from Nigerian Medicinal Plants
Source: PLoS Negl Trop Dis. 2012 Jul 24;6(7):e1727. doi: 10.1371/journal.pntd.0001727 (PMC3404109; doi:10.1371/journal.pntd.0001727)
Supplement: Table S10 — Lowest-energy docking energies (kcal/mol) for Lawsonia inermis phytochemicals with Trypanosoma brucei protein targets. (DOCX) [file pntd.0001727.s010.docx]

**Table S10.** Lowest-energy docking energies (kcal/mol) for *Lawsonia inermis* phytochemicals with *Trypanosoma brucei* protein targets.^a^

| Compound | Rhodesain | TbAK | TbPTR1 | TbDHFR | TbTR | TbCatB | TbHSP90 | TbCYP51 | TbNH | TbTIM | TbNDRT | TbUDPGE | TbODC |
| --- | --- | --- | --- | --- | --- | --- | --- | --- | --- | --- | --- | --- | --- |
|   Isoplumbagin | -15.5 | -17.2 | **-19.8** | -17.0 | -17.2 | -13.7 | -18.4 | -16.7 | -16.7 | **-19.7** | -13.6 | -18.8 | -16.7 |
|   Lacoumarin | -19.3 | -20.5 | **-23.0** | -19.7 | -18.6 | -15.9 | -19.7 | -18.6 | -19.0 | -21.6 | -19.6 | -20.3 | -19.4 |
|   Lawnermis acid | -10.5 | **-23.6** | -11.8 | -20.7 | -17.9 | -14.8 | -15.0 | **-25.3** | -17.3 | -13.7 | -9.7 | -19.4 | -19.0 |
|   Lawnermis acid methyl ester | -6.7 | -24.4 | -14.7 | -21.8 | -20.1 | -15.8 | -14.7 | **-28.2** | -18.4 | -17.1 | -15.9 | -23.8 | -20.9 |
|   Lawsaritol | -16.8 | -24.2 | -24.3 | -24.0 | -23.4 | -19.4 | -19.7 | **-26.4** | **-26.8** | -26.1 | -21.6 | **-26.6** | -24.9 |
|   Lawsaritol A | -16.4 | -26.4 | -24.9 | -22.1 | -23.1 | -20.3 | -19.8 | **-28.3** | -26.4 | -19.3 | -21.8 | -27.1 | -24.9 |
|   Lawsonadeem | -19.2 | **-25.5** | -14.9 | -18.8 | -22.4 | -18.6 | -19.3 | -20.8 | -23.7 | **-26.3** | -16.5 | -24.4 | -19.3 |
|   Lawsone | -15.1 | -18.1 | -18.2 | -16.2 | -16.8 | -13.1 | -16.3 | -14.7 | -15.0 | **-18.7** | -15.4 | -17.2 | -16.2 |
|   Lawsonicin | -19.3 | -26.5 | -28.5 | -25.6 | -27.2 | -19.9 | -24.4 | -27.3 | -28.5 | -26.7 | -23.2 | **-30.6** | -26.8 |
|   Laxanthone I | -20.0 | -22.8 | **-25.2** | -20.3 | -21.6 | -16.4 | -19.6 | -20.0 | -22.8 | -23.7 | -20.9 | -22.5 | -22.0 |
|   Laxanthone II | -25.3 | -26.5 | **-29.4** | -26.0 | -25.6 | -19.2 | -24.2 | -23.9 | -28.5 | -27.2 | -25.6 | -28.1 | -25.3 |
|   Laxanthone III | -20.8 | -24.5 | **-27.7** | -23.8 | -23.8 | -18.5 | -21.4 | -23.3 | -25.9 | -26.1 | -25.1 | -25.7 | -24.3 |
|   Wallichianol | -13.4 | -22.7 | -19.7 | -20.5 | -20.6 | -17.2 | -18.1 | **-27.0** | -20.0 | no dock | -7.8 | -24.2 | -23.4 |

^a^Ligands showing selective (significantly stronger docking than average for all proteins) docking energies are highlighted in **blue bold**.
