# Supplementary material for: In-silico Investigation of Antitrypanosomal Phytochemicals from Nigerian Medicinal Plants
Source: PLoS Negl Trop Dis. 2012 Jul 24;6(7):e1727. doi: 10.1371/journal.pntd.0001727 (PMC3404109; doi:10.1371/journal.pntd.0001727)
Supplement: Table S11 — Lowest-energy docking energies (kcal/mol) for Morinda lucida phytochemicals with Trypanosoma brucei protein targets. (DOCX) [file pntd.0001727.s011.docx]

**Table S11.** Lowest-energy docking energies (kcal/mol) for *Morinda lucida* phytochemicals with *Trypanosoma brucei* protein targets.^a^

| Compound | Rhodesain | TbAK | TbPTR1 | TbDHFR | TbTR | TbCatB | TbHSP90 | TbCYP51 | TbNH | TbTIM | TbNDRT | TbUDPGE | TbODC |
| --- | --- | --- | --- | --- | --- | --- | --- | --- | --- | --- | --- | --- | --- |
|   1-Hydroxy-2-methylanthraquinone | -18.6 | -19.2 | -22.3 | -16.7 | -19.8 | -14.4 | -20.2 | -15.7 | -19.6 | -21.8 | -14.9 | -21.1 | -19.6 |
|   2-Formyl-3-hydroxyanthraquinone | -17.6 | -20.6 | **-23.7** | -19.3 | -21.8 | -18.3 | -19.4 | -18.8 | -21.3 | **-23.8** | -17.5 | -22.5 | -19.8 |
|   2-Formylanthraquinone | -17.1 | -20.4 | **-23.4** | -17.5 | -20.1 | -16.7 | -19.3 | -18.7 | -20.0 | **-23.5** | -16.4 | -21.4 | -19.3 |
|   2-Hydroxy-1-methoxyanthraquinone | -17.1 | -20.1 | -22.7 | -18.3 | -20.8 | -15.6 | -21.2 | -16.4 | -19.8 | -21.4 | -17.2 | -21.7 | -18.2 |
|   2-Hydroxy-3-hydroxymethylanthraquinone | -17.4 | -20.5 | -23.8 | -19.1 | -21.8 | -18.4 | -19.1 | -18.4 | -21.8 | **-24.0** | -18.0 | -23.4 | -20.5 |
|   1,1'-Di-*O*-methyllucidin | -18.2 | -23.2 | **-25.2** | -20.6 | -22.1 | -16.2 | -20.4 | -20.0 | -23.0 | -22.8 | -14.9 | **-25.4** | -21.4 |
|   5-*O*-Methylmorindone | -19.9 | -20.7 | **-24.5** | -18.4 | -20.8 | -17.3 | -20.1 | -17.2 | -21.5 | -22.2 | -16.5 | -22.9 | -20.3 |
|   Munjistin | -18.9 | -21.9 | **-25.5** | -18.9 | -21.9 | -18.8 | -20.4 | -18.8 | -22.6 | -23.4 | -16.0 | -24.7 | -20.9 |
|   Nordamnacanthal | -19.4 | -21.1 | **-25.3** | -19.5 | -22.0 | -17.8 | -19.2 | -17.9 | -22.2 | **-24.7** | -16.1 | -23.5 | -20.8 |
|   Oleanolic acid | -4.9 | -22.0 | -15.4 | -20.5 | -15.6 | -19.2 | -17.0 | -24.0 | -17.2 | -11.3 | -10.5 | -22.3 | **-25.9** |
|   Oruwacin | -25.7 | -28.8 | **-32.4** | -27.7 | -27.6 | -24.7 | -27.5 | -29.2 | **-31.6** | -29.2 | -17.4 | -30.4 | -27.2 |
|   Oruwal | -16.4 | -21.3 | **-25.0** | -18.6 | -21.1 | -17.9 | -20.0 | -18.6 | -20.6 | -22.8 | -14.8 | -23.0 | -19.6 |
|   Oruwalol | -17.3 | -22.5 | **-25.7** | -19.6 | -22.7 | -18.7 | -22.2 | -18.9 | -21.6 | -21.1 | -15.8 | -23.8 | -19.3 |
|   Rubiadin | -18.7 | -19.9 | **-23.1** | -18.4 | -21.1 | -15.7 | -18.4 | -17.3 | -21.0 | **-23.3** | -16.0 | -22.6 | -20.0 |
|   Soranjidiol | -19.3 | -20.4 | -23.6 | -18.3 | -20.3 | -15.8 | -18.7 | -16.7 | -20.9 | **-23.6** | -17.0 | -21.7 | -20.3 |
|   Ursolic acid | -12.6 | -22.9 | -13.9 | -19.9 | -19.4 | -17.5 | -17.6 | **-24.3** | -18.2 | -11.2 | -10.6 | -21.3 | -23.1 |

^a^Ligands showing selective (significantly stronger docking than average for all proteins) docking energies are highlighted in **blue bold**.
