# Supplementary material for: In-silico Investigation of Antitrypanosomal Phytochemicals from Nigerian Medicinal Plants
Source: PLoS Negl Trop Dis. 2012 Jul 24;6(7):e1727. doi: 10.1371/journal.pntd.0001727 (PMC3404109; doi:10.1371/journal.pntd.0001727)
Supplement: Table S12 — Lowest-energy docking energies (kcal/mol) for Morinda morindoides phytochemicals with Trypanosoma brucei protein targets. (DOCX) [file pntd.0001727.s012.docx]

**Table S12.** Lowest-energy docking energies (kcal/mol) for *Morinda morindoides* phytochemicals with *Trypanosoma brucei* protein targets.^a^

| Compound | Rhodesain | TbAK | TbPTR1 | TbDHFR | TbTR | TbCatB | TbHSP90 | TbCYP51 | TbNH | TbTIM | TbNDRT | TbUDPGE | TbODC |
| --- | --- | --- | --- | --- | --- | --- | --- | --- | --- | --- | --- | --- | --- |
|   Acetylgaertneroside | -27.2 | -37.4 | -36.5 | **-37.7** | -29.7 | -28.5 | -32.9 | -35.5 | -32.6 | -30.8 | -15.8 | -30.9 | -34.5 |
|   Apigenin | -21.2 | -22.1 | **-24.6** | -20.7 | -23.4 | -17.8 | -22.8 | -21.0 | -22.9 | -22.8 | -23.2 | -24.1 | -21.5 |
|   Apigenin-7-*O*-glucoside (= Cosmosiin) | -25.8 | -30.7 | **-32.4** | -28.9 | -27.7 | -24.4 | -29.3 | -30.1 | -31.1 | -28.5 | -23.6 | **-33.0** | -28.0 |
|   Chrysoeriol | -23.4 | -25.6 | **-27.7** | -22.4 | -23.3 | -20.8 | -24.9 | -22.6 | -23.9 | -24.4 | -26.0 | -26.7 | -23.2 |
| Chrysoeriol 7-neohesperidoside | -28.4 | -35.6 | -33.3 | -34.6 | -33.1 | -27.3 | -32.9 | -33.2 | -31.7 | -31.9 | -14.2 | **-37.2** | -33.0 |
|   Dehydroepoxymethoxygaertneroside | -25.8 | -36.7 | -35.2 | -37.3 | -30.7 | -29.5 | -35.0 | -32.6 | **-40.6** | -35.9 | -23.2 | -34.3 | -35.6 |
|   Dehydrogaertneroside | -28.5 | -35.7 | -34.8 | -37.1 | -32.3 | -31.3 | -32.5 | -35.2 | -38.1 | -32.1 | -12.4 | -37.4 | -35.5 |
|   Dehydromethoxygaertneroside | -27.2 | -35.0 | -35.0 | **-38.9** | -34.9 | -33.1 | -34.1 | -36.4 | -37.8 | -34.9 | -25.6 | -30.4 | -36.3 |
|   Epoxygaertneroside | -24.9 | **-38.4** | -31.5 | **-37.4** | -30.5 | -28.2 | -32.8 | -33.5 | -35.1 | -32.1 | -21.7 | -31.4 | -34.2 |
|   Epoxymethoxygaertneroside | -27.3 | -35.9 | -35.6 | -37.6 | -31.3 | -33.0 | -34.1 | -36.3 | -37.6 | -34.2 | -11.3 | -33.6 | -35.3 |
|   Gaertneric acid | -23.8 | -32.9 | -32.7 | **-37.2** | -29.1 | -30.8 | -33.4 | -35.0 | -35.9 | -31.0 | -19.0 | -32.7 | -32.4 |
|   Gaertneroside | -27.3 | -35.7 | -34.5 | **-37.8** | -29.4 | -29.2 | -32.1 | -34.7 | -35.5 | -31.9 | -16.7 | -32.4 | -33.2 |
|   Kaempferol | -23.8 | -24.7 | **-25.6** | -21.4 | -22.1 | -18.6 | -23.8 | -21.8 | -23.4 | -24.6 | -24.3 | -25.0 | -21.5 |
|   Kaempferol-3-*O*-rhamnoside (= Azfelin) | -22.3 | -30.5 | **-31.2** | -28.1 | -26.5 | -23.3 | -28.8 | -26.4 | -30.4 | -27.7 | -23.0 | -28.3 | -26.5 |
|   Kaempferol-3-*O*-rutinoside (= Nicotiflorin) | -25.7 | -35.9 | -33.0 | -36.5 | -32.7 | -30.9 | -30.8 | -32.8 | -34.5 | -30.3 | -16.6 | -33.3 | **-37.0** |
|   Luteolin | -23.0 | -24.7 | -25.4 | -20.6 | -23.3 | -18.6 | -23.8 | -22.2 | -24.5 | -24.2 | -24.6 | -25.7 | -22.7 |
|   Luteolin-7-*O*-glucoside (= Glucoluteolin) | -25.8 | -32.1 | -32.5 | -29.4 | -29.3 | -24.3 | -30.9 | -31.7 | -33.9 | -29.4 | -28.1 | **-34.7** | -30.1 |
|   Methoxygaertneroside | -26.5 | -33.3 | -34.3 | **-36.6** | -28.5 | -28.9 | -33.4 | -32.7 | -35.8 | -32.1 | -17.5 | -31.0 | -34.6 |
| Morindaoside | -28.4 | **-39.2** | **-39.2** | -29.3 | -36.8 | -34.8 | -33.6 | -37.8 | -37.6 | -34.4 | no dock | -35.8 | -38.6 |
|   Quercetin | -22.7 | -25.9 | **-26.1** | -21.7 | -23.9 | -19.8 | -24.7 | -22.2 | -25.1 | -25.4 | -25.6 | **-26.5** | -22.8 |
|   Quercetin-3-*O*-rhamnoside (= Quercitrin) | -23.0 | -29.6 | **-32.1** | -28.2 | -27.6 | -23.5 | -29.6 | -28.1 | **-32.4** | -29.1 | -23.4 | -28.1 | -27.7 |
|   Quercetin-3-*O*-rutinoside (= Rutoside) | -26.1 | -34.0 | -33.3 | -36.9 | -32.3 | -32.1 | -32.6 | -34.4 | -37.3 | -31.3 | -16.5 | -33.2 | -36.9 |
|   Quercetin 4',7-dimethylether (= Ombuin) | -22.4 | **-27.6** | **-27.4** | -22.6 | -23.6 | -22.5 | -25.4 | -23.3 | -25.6 | -26.0 | -24.0 | **-27.5** | -23.7 |

^a^Ligands showing selective (significantly stronger docking than average for all proteins) docking energies are highlighted in **blue bold**.
