# Supplementary material for: In-silico Investigation of Antitrypanosomal Phytochemicals from Nigerian Medicinal Plants
Source: PLoS Negl Trop Dis. 2012 Jul 24;6(7):e1727. doi: 10.1371/journal.pntd.0001727 (PMC3404109; doi:10.1371/journal.pntd.0001727)
Supplement: Table S13 — Lowest-energy docking energies (kcal/mol) for Nauclea latifolia phytochemicals with Trypanosoma brucei protein targets. (DOCX) [file pntd.0001727.s013.docx]

**Table S13.** Lowest-energy docking energies (kcal/mol) for *Nauclea latifolia* phytochemicals with *Trypanosoma brucei* protein targets.^a^

| Compound | Rhodesain | TbAK | TbPTR1 | TbDHFR | TbTR | TbCatB | TbHSP90 | TbCYP51 | TbNH | TbTIM | TbNDRT | TbUDPGE | TbODC |
| --- | --- | --- | --- | --- | --- | --- | --- | --- | --- | --- | --- | --- | --- |
|   10-Hydroxyangustine | -19.5 | -25.7 | -28.3 | -20.1 | -25.1 | -19.7 | -23.3 | -20.8 | -26.0 | -23.4 | -18.0 | -25.9 | -23.1 |
|   10-Hydroxystrictosamide | -22.7 | -26.8 | -24.5 | -25.2 | -28.2 | -23.3 | -24.1 | -28.8 | -23.9 | -25.3 | -23.6 | **-31.3** | **-29.1** |
|   Angustine | -19.4 | -24.7 | **-28.4** | -19.4 | -24.0 | -19.4 | -23.3 | -19.6 | -24.5 | -23.3 | -17.2 | -23.6 | -21.4 |
|   Cadambine | -23.0 | -28.6 | -28.2 | -30.5 | -30.4 | -23.2 | -27.9 | -29.2 | -26.1 | -20.8 | no dock | **-32.6** | -30.7 |
|   Decarbomethoxynauclechine | -16.2 | **-24.7** | **-24.8** | -19.3 | -21.6 | -16.9 | -21.7 | -21.6 | **-24.9** | -23.3 | -17.4 | -23.1 | -21.1 |
|   Dihydrocadambine | -27.1 | -32.8 | -28.7 | -26.1 | -27.4 | -23.8 | -28.2 | -29.7 | -32.1 | -19.4 | -16.8 | **-35.8** | -28.8 |
|   Fucoquinovic acid | -3.1 | -23.3 | -15.9 | -15.1 | -22.6 | -22.4 | -17.8 | -18.3 | -10.1 | no dock | no dock | -13.7 | **-24.5** |
|   Naucleamide A | -20.6 | -22.7 | -26.3 | -21.8 | -25.8 | -17.1 | -25.1 | -21.9 | -25.6 | -23.7 | -21.3 | -26.3 | -23.5 |
|   Naucleamide B | -20.5 | -27.6 | **-29.5** | -22.8 | -24.4 | -19.2 | -22.5 | -22.3 | -27.1 | -24.1 | -25.2 | -25.4 | -23.5 |
|   Naucleamide C | -21.1 | **-26.4** | -25.1 | -20.8 | -22.5 | -17.9 | -22.7 | -20.4 | -24.0 | -23.5 | -15.6 | -23.8 | -22.7 |
|   Naucleamide D | -19.3 | -25.5 | -27.4 | -21.3 | -22.7 | -14.5 | -24.3 | -23.0 | -26.5 | -24.6 | -23.9 | -26.9 | -25.3 |
|   Naucleamide E | -16.6 | -23.6 | -25.5 | -23.4 | -22.7 | -15.4 | -20.8 | -21.8 | **-26.6** | -23.2 | -15.4 | -23.7 | -23.3 |
|   Naucleamide F | -22.3 | **-27.5** | -21.6 | -21.8 | -26.8 | -21.6 | -23.1 | -27.0 | -15.1 | -26.2 | -25.8 | -27.7 | -25.3 |
|  |  |  |  |  |  |  |  |  |  |  |  |  |  |
|   Nauclefine | -15.2 | -23.6 | -24.5 | -16.7 | -22.3 | -18.2 | -20.7 | -19.7 | -23.4 | -23.2 | -19.3 | -24.1 | -20.0 |
|   Nauclefoline | -19.6 | -26.0 | -26.7 | -23.1 | -22.8 | -20.4 | -25.4 | -25.6 | -26.4 | -24.2 | -20.1 | -25.4 | -25.6 |
|   Nauclefolinine | -15.7 | **-28.9** | -21.7 | -18.7 | -23.4 | -19.7 | -24.2 | -21.3 | -24.7 | -23.1 | -17.3 | -25.8 | -21.7 |
|   Naucleindinal | -19.2 | -23.5 | **-27.0** | -19.6 | -22.0 | -18.6 | -19.7 | -21.4 | -24.2 | -23.4 | -17.5 | -21.2 | -23.1 |
|   Naucletine | -19.9 | -24.5 | **-29.2** | -20.2 | -26.2 | -19.6 | -24.1 | -20.5 | -25.6 | -23.7 | -18.9 | -23.7 | -22.3 |
|   Naufoline | -21.2 | -24.2 | -24.1 | -17.9 | -22.0 | -17.6 | -21.7 | -21.2 | -24.5 | -23.9 | -17.1 | -24.1 | -20.8 |
|   Naulafine | -19.8 | -25.5 | **-26.9** | -21.0 | -25.5 | -18.6 | -22.8 | -19.2 | -23.9 | -26.2 | -16.3 | -24.7 | -22.0 |
|   Rhamnoquinovic acid | no dock | -24.2 | -14.4 | -15.0 | -23.0 | -22.7 | -20.9 | -22.5 | -12.4 | -13.9 | no dock | -10.0 | -24.5 |
|   Rotundic acid | -0.3 | -20.3 | -5.3 | -17.2 | -18.6 | -17.5 | -11.3 | -26.0 | -18.5 | no dock | -10.6 | **-27.3** | -21.9 |
|   Strictosamide | -21.7 | -27.0 | -25.6 | -24.9 | -27.3 | -23.2 | -24.0 | **-29.8** | -26.8 | -23.1 | -18.0 | -29.4 | -28.0 |
|   Tetrahydrodesoxycordifoline | -28.6 | -35.2 | -33.3 | -33.8 | -29.4 | -25.5 | -31.7 | **-36.3** | -33.5 | -26.8 | -13.0 | -32.2 | -33.4 |

^a^Ligands showing selective (significantly stronger docking than average for all proteins) docking energies are highlighted in **blue bold**.
