# Supplementary material for: In-silico Investigation of Antitrypanosomal Phytochemicals from Nigerian Medicinal Plants
Source: PLoS Negl Trop Dis. 2012 Jul 24;6(7):e1727. doi: 10.1371/journal.pntd.0001727 (PMC3404109; doi:10.1371/journal.pntd.0001727)
Supplement: Table S14 — Lowest-energy docking energies (kcal/mol) for Newbouldia laevis phytochemicals with Trypanosoma brucei protein targets. (DOCX) [file pntd.0001727.s014.docx]

**Table S14.** Lowest-energy docking energies (kcal/mol) for *Newbouldia laevis* phytochemicals with *Trypanosoma brucei* protein targets.^a^

| Compound | Rhodesain | TbAK | TbPTR1 | TbDHFR | TbTR | TbCatB | TbHSP90 | TbCYP51 | TbNH | TbTIM | TbNDRT | TbUDPGE | TbODC |
| --- | --- | --- | --- | --- | --- | --- | --- | --- | --- | --- | --- | --- | --- |
|   2-Isopropenylnaphtho[2,3-*b*]furan-4,9-dione | -20.2 | -21.7 | -23.3 | -19.8 | -21.4 | -15.4 | -21.3 | -19.3 | -21.8 | **-24.8** | -17.2 | -19.9 | -19.5 |
|   2,3-Dihydro-6-hydroxy-2-isopropenylbenzofuran | -16.6 | -17.7 | -19.1 | -16.9 | -18.3 | -13.2 | -19.5 | -17.5 | -17.4 | -20.4 | -17.8 | -17.7 | -20.6 |
|   3-Hydroxy-5-methoxydehydroiso-α-lapachone | -19.1 | -24.3 | -23.9 | -19.9 | -22.6 | -17.6 | -22.8 | -20.7 | -22.7 | **-26.4** | -18.7 | -23.1 | -22.8 |
|   4*'*-Hydroxynewbouldine | -17.4 | -19.4 | -20.8 | -19.1 | -19.3 | -15.9 | -19.8 | -18.7 | -20.3 | -20.3 | -20.7 | -19.8 | -18.6 |
|   4*'*Hydroxywithasomnine | -18.1 | -20.6 | -21.2 | -18.7 | -20.4 | -17.8 | -21.2 | -18.9 | -19.5 | **-23.1** | -20.6 | -20.8 | -19.6 |
|   4*'*-Methoxynewbouldine | -18.3 | -20.3 | **-21.8** | -20.2 | -19.8 | -17.2 | -17.9 | -20.3 | -21.1 | -21.5 | -19.9 | -20.9 | -19.3 |
|   4*'*-Methoxywithasomnine | -18.8 | -21.1 | **-22.1** | -20.7 | -20.3 | -17.3 | -18.8 | -20.3 | -20.5 | **-22.0** | -18.7 | -21.2 | -20.5 |
|   5-Hydroxy-2-isopropenylnaphtho[2,3-*b*]furan-4,9-dione | -20.7 | -22.2 | -23.8 | -21.2 | -21.7 | -21.3 | -21.9 | -20.2 | -22.7 | **-24.3** | -18.2 | -21.1 | -22.9 |
|   5-Hydroxy-7-methoxydehydroiso-α-lapachone | -19.8 | -23.6 | -24.7 | -23.7 | -23.5 | -19.1 | -22.2 | -21.5 | -24.2 | **-27.1** | -18.3 | -24.1 | -21.7 |
|   5-hydroxydehydroiso-α-lapachone | -19.2 | -21.8 | -23.6 | -20.7 | -21.2 | -18.3 | -22.7 | -19.5 | -22.0 | **-26.2** | -17.8 | -20.8 | -21.3 |
|   5,7-Dihydroxydehydroiso-α-lapachone | -19.4 | -22.3 | -24.7 | -22.3 | -23.1 | -18.7 | -24.4 | -19.7 | -23.7 | **-26.6** | -18.8 | -22.6 | -21.3 |
|   6-Hydroxydehydroiso-α-lapachone | -19.4 | -22.4 | **-25.2** | -20.6 | -22.7 | -17.4 | -22.3 | -20.7 | -22.4 | **-27.1** | -20.6 | -22.9 | -22.1 |
|   7-Hydroxy-2-isopropenylnaphtho[2,3-*b*]furan-4,9-dione | -20.3 | -22.5 | **-25.9** | -21.1 | -22.4 | -15.3 | -22.9 | -20.2 | -23.3 | **-26.1** | -18.4 | -21.7 | -21.0 |
|   7-Hydroxydehydroiso-α-lapachone | -17.3 | -21.9 | **-24.9** | -20.5 | -21.8 | -16.0 | -23.0 | -20.2 | -22.6 | **-26.2** | -19.5 | -22.0 | -22.6 |
|   Newbouldiaquinone | -17.9 | -25.1 | -21.5 | -22.1 | **-25.4** | -19.1 | **-25.7** | -22.2 | -24.4 | -24.0 | -16.5 | -20.8 | -22.9 |
|   Newbouldiaquinone A | -21.2 | **-30.0** | -26.5 | -25.7 | -26.1 | -19.7 | -22.5 | -26.2 | **-29.4** | -27.7 | -22.2 | -27.2 | -25.5 |
|   Newbouldine | -16.7 | -17.9 | **-20.9** | -17.2 | -18.9 | -15.1 | -20.1 | -18.1 | -18.9 | **-20.6** | -18.4 | -17.6 | -17.8 |
|   Withasomnine | -17.4 | -18.6 | -20.6 | -17.5 | -18.8 | -16.2 | -20.2 | -19.9 | -18.5 | **-22.8** | -18.0 | -19.3 | -19.0 |

^a^Ligands showing selective (significantly stronger docking than average for all proteins) docking energies are highlighted in **blue bold**.
