# Supplementary material for: In-silico Investigation of Antitrypanosomal Phytochemicals from Nigerian Medicinal Plants
Source: PLoS Negl Trop Dis. 2012 Jul 24;6(7):e1727. doi: 10.1371/journal.pntd.0001727 (PMC3404109; doi:10.1371/journal.pntd.0001727)
Supplement: Table S15 — Lowest-energy docking energies (kcal/mol) for Physalis angulata phytochemicals with Trypanosoma brucei protein targets. (DOCX) [file pntd.0001727.s015.docx]

**Table S15.** Lowest-energy docking energies (kcal/mol) for *Physalis angulata* phytochemicals with *Trypanosoma brucei* protein targets.^a^

| Compound | Rhodesain | TbAK | TbPTR1 | TbDHFR | TbTR | TbCatB | TbHSP90 | TbCYP51 | TbNH | TbTIM | TbNDRT | TbUDPGE | TbODC |
| --- | --- | --- | --- | --- | --- | --- | --- | --- | --- | --- | --- | --- | --- |
|   14-Hydroxyixocarpanolide | -7.0 | -23.1 | -20.6 | -21.9 | -25.0 | -20.2 | -20.6 | **-29.9** | -20.8 | -22.1 | -21.6 | -27.5 | -24.1 |
|   24,25-Epoxywithanolide D | -9.3 | -26.5 | -25.8 | -27.5 | -26.6 | -22.2 | -22.4 | -28.6 | -25.0 | -18.0 | -21.9 | -27.8 | -28.4 |
|   Phygrine | -20.7 | -24.8 | -23.9 | -22.8 | -21.8 | -17.7 | -23.0 | -21.2 | -24.4 | **-25.5** | -23.8 | -23.3 | -21.6 |
|   Physagulin A | -15.0 | -27.5 | -25.8 | -24.1 | -23.8 | -22.3 | -25.5 | -26.5 | -18.7 | -19.1 | -19.5 | -28.1 | **-28.2** |
|   Physagulin B | -15.9 | -26.6 | -24.8 | -23.8 | -24.3 | -21.5 | -24.7 | -24.0 | -23.4 | -24.0 | -17.2 | **-29.3** | -26.8 |
|   Physagulin C | -22.6 | -26.9 | -25.3 | -22.9 | -25.7 | -20.7 | -25.0 | -27.4 | -22.7 | -10.2 | -13.5 | -27.0 | -27.5 |
|   Physagulin F | -16.8 | -26.2 | -23.5 | -22.4 | -24.4 | -20.1 | -23.9 | -25.3 | -20.7 | -9.7 | -14.7 | -25.0 | -26.7 |
|   Physagulin H | -19.1 | -26.7 | -22.9 | -25.9 | -24.1 | -20.4 | -26.6 | -27.6 | -21.0 | -16.8 | -14.0 | -25.2 | -26.7 |
|   Physagulin I | -20.6 | -25.7 | -22.4 | -22.6 | -22.7 | -20.5 | -23.5 | -25.4 | -19.7 | -10.6 | -15.7 | -25.9 | -25.4 |
|   Physagulin J | -15.9 | -27.3 | -18.8 | -24.9 | -23.6 | -20.7 | -20.5 | **-29.0** | -24.4 | -18.7 | -15.6 | -27.7 | -26.7 |
|   Physagulin K | -17.9 | -25.5 | -20.3 | -24.1 | -19.3 | -24.8 | -19.9 | **-27.8** | -15.3 | -23.2 | -15.5 | -27.1 | -25.4 |
|   Physagulin L | -15.9 | -27.1 | -22.7 | -24.6 | -21.6 | -24.6 | -20.2 | **-29.0** | -21.0 | -22.2 | -3.6 | -26.7 | -25.4 |
|   Physagulin L*'* | -19.7 | **-29.5** | -24.9 | -24.8 | -25.2 | -22.0 | -27.2 | -26.3 | -25.8 | -25.6 | -19.6 | -28.3 | **-29.3** |
|   Physagulin M | -15.8 | -25.9 | -21.2 | -22.4 | -24.6 | -21.4 | -24.3 | -26.0 | -25.2 | -12.8 | -13.7 | -26.5 | -24.5 |
|   Physagulin M*'* | -17.7 | -26.8 | -24.1 | -26.1 | -24.5 | -22.8 | -21.8 | -26.3 | -22.6 | -5.8 | -12.1 | -26.3 | -26.4 |
|   Physagulin N | -20.2 | -23.5 | -21.5 | -22.7 | -24.5 | -24.6 | -23.2 | -27.8 | -23.2 | -16.1 | -13.9 | **-31.0** | -25.8 |
|   Physagulin N*'* | -17.0 | -23.6 | -20.8 | -21.4 | -23.2 | -22.8 | -17.7 | **-25.8** | -15.2 | no dock | -15.4 | -24.8 | -22.0 |
|   Physagulin O | -19.5 | -25.0 | -18.7 | -23.7 | -21.4 | -19.9 | -25.0 | -25.7 | -20.1 | -16.6 | -6.2 | -26.0 | **-26.6** |
|   Physalin A | -14.9 | -23.5 | -16.5 | -20.8 | -19.4 | -21.5 | -16.8 | -20.0 | -8.9 | -5.3 | -17.5 | -21.5 | **-23.9** |
|   Physalin B | -18.1 | **-24.8** | -18.2 | -19.2 | -18.5 | -19.6 | -20.6 | **-25.3** | -13.8 | -8.7 | -12.5 | -21.0 | -20.9 |
|  |  |  |  |  |  |  |  |  |  |  |  |  |  |
|   Physalin D | -2.6 | -22.9 | -17.0 | -19.5 | -18.9 | -20.2 | -18.0 | **-26.7** | -14.6 | -13.0 | -7.7 | -22.8 | -20.2 |
|   Physalin E | -1.8 | -21.1 | -17.9 | -17.9 | -18.8 | -21.0 | -8.6 | -21.5 | -3.9 | no dock | -13.8 | -22.2 | -21.0 |
|   Physalin F | -18.9 | **-24.4** | -17.1 | -19.7 | -18.6 | -21.9 | -13.7 | **-24.8** | -6.5 | no dock | -14.0 | -21.4 | -19.7 |
|   Physalin G | -22.2 | **-24.5** | -17.7 | -20.5 | -23.6 | -22.8 | -19.0 | -22.6 | -17.5 | -16.3 | -11.6 | -23.0 | -23.0 |
|   Physalin H | -4.7 | -22.9 | -17.4 | -20.0 | -18.4 | -20.8 | -14.1 | **-26.4** | no dock | -8.9 | -8.7 | -21.5 | -21.2 |
|   Physalin I | -6.2 | -22.4 | -15.3 | -20.4 | -18.8 | -19.9 | -13.7 | **-25.6** | no dock | -10.9 | no dock | -11.3 | -21.4 |
|   Physalin J | -3.6 | -21.8 | -18.8 | -21.2 | -19.3 | -22.4 | -15.8 | **-25.1** | -12.6 | -11.3 | -14.7 | -19.5 | -22.1 |
|   Physalin K | -13.1 | -21.1 | -17.4 | -18.9 | -19.1 | -19.5 | -14.5 | **-21.0** | -8.2 | -10.3 | -11.5 | -20.4 | -19.6 |
|   Physalin U | -17.5 | -23.8 | -17.2 | -21.2 | -20.0 | -23.9 | -12.8 | -23.9 | -6.2 | no dock | -8.3 | -24.8 | -21.5 |
|   Physalin V | -21.2 | **-25.5** | -17.1 | -20.2 | -20.4 | -22.0 | -22.8 | -22.0 | -7.2 | -16.0 | -11.8 | -21.7 | -23.1 |
|   Physalin W | -20.0 | -22.6 | -15.8 | -21.8 | -19.6 | -22.3 | -10.1 | **-26.2** | -8.6 | no dock | -5.3 | -23.8 | -22.3 |
|   Physangulide | -16.9 | -24.2 | -20.4 | -22.7 | -24.6 | -23.6 | -21.3 | **-26.1** | -24.1 | -23.2 | -22.4 | **-25.9** | -22.9 |
|   Physanolide A | -19.7 | -23.3 | -21.3 | -27.3 | -22.6 | -21.8 | -19.8 | -24.7 | -16.6 | -16.8 | -11.3 | -26.4 | -24.8 |
|   Vamonolide | -11.8 | -23.1 | -20.6 | -20.9 | -23.0 | -19.8 | -20.6 | **-28.0** | -18.4 | -20.1 | -21.6 | **-26.5** | -23.5 |
|   Withangulatin A | -12.2 | -25.9 | -26.1 | -24.5 | -25.6 | -22.2 | -21.8 | -27.0 | -23.7 | -19.8 | -19.5 | -26.7 | **-29.5** |
|   Withangulatin B | -8.8 | -23.7 | -22.1 | -22.6 | -23.3 | -22.8 | -22.1 | **-27.5** | -13.3 | -21.4 | -20.0 | **-27.8** | -22.9 |
|   Withangulatin C | -7.3 | -25.8 | -22.7 | -23.8 | -23.3 | -22.7 | -21.6 | -27.5 | -18.5 | -14.0 | -21.0 | **-29.0** | -24.7 |
|   Withangulatin D | -0.4 | -23.4 | -21.7 | -23.3 | -23.9 | -22.4 | -21.5 | -26.5 | -10.0 | -4.0 | -12.3 | -24.5 | -26.6 |
|   Withangulatin E | -12.1 | -23.2 | -21.1 | -21.0 | -21.3 | -22.4 | -17.9 | **-28.4** | -16.7 | -24.5 | -19.3 | -26.6 | -24.2 |
|   Withangulatin F | -9.5 | -23.7 | -21.8 | -22.7 | -23.4 | -21.2 | -20.4 | **-28.1** | -21.1 | -23.7 | -24.6 | -25.6 | -26.1 |
|   Withangulatin G | -9.4 | -24.2 | -21.3 | -22.5 | -22.8 | -24.3 | -21.2 | -24.4 | -7.3 | -1.4 | -15.2 | **-33.1** | -23.7 |
|   Withangulatin H | -7.1 | -22.5 | -16.4 | -21.5 | -24.8 | -19.5 | -19.8 | **-29.6** | -19.6 | -24.2 | -17.9 | -26.4 | -22.1 |
|   Withangulatin I | -19.2 | -24.9 | -22.9 | -23.2 | -25.2 | -25.1 | -27.0 | **-29.0** | -21.5 | -12.2 | -19.7 | **-28.9** | -27.3 |

^a^Ligands showing selective (significantly stronger docking than average for all proteins) docking energies are highlighted in **blue bold**.
