# Supplementary material for: In-silico Investigation of Antitrypanosomal Phytochemicals from Nigerian Medicinal Plants
Source: PLoS Negl Trop Dis. 2012 Jul 24;6(7):e1727. doi: 10.1371/journal.pntd.0001727 (PMC3404109; doi:10.1371/journal.pntd.0001727)
Supplement: Table S16 — Lowest-energy docking energies (kcal/mol) for Picralima nitida phytochemicals with Trypanosoma brucei protein targets. (DOCX) [file pntd.0001727.s016.docx]

**Table S16.** Lowest-energy docking energies (kcal/mol) for *Picralima nitida* phytochemicals with *Trypanosoma brucei* protein targets.^a^

| Compound | Rhodesain | TbAK | TbPTR1 | TbDHFR | TbTR | TbCatB | TbHSP90 | TbCYP51 | TbNH | TbTIM | TbNDRT | TbUDPGE | TbODC |
| --- | --- | --- | --- | --- | --- | --- | --- | --- | --- | --- | --- | --- | --- |
|   Ajmalicine | -23.1 | -26.3 | **-30.3** | -23.0 | -23.0 | -21.4 | -25.7 | -21.9 | -25.4 | -21.6 | -21.7 | -25.7 | -24.8 |
|   Akuammicine | -18.2 | -21.8 | -23.1 | -22.2 | -21.3 | -19.3 | -19.2 | -21.6 | -20.8 | -19.9 | -18.9 | -23.5 | -22.2 |
|   Akuammidine | -12.1 | -21.5 | -21.7 | -18.0 | -21.2 | -19.4 | -16.7 | -22.5 | -20.0 | -20.2 | -11.7 | -23.5 | -17.7 |
|   Akuammigine | -18.0 | -24.9 | **-28.1** | -20.2 | -22.2 | -19.6 | -24.8 | -24.8 | **-28.1** | -21.6 | -19.0 | -29.5 | -24.5 |
|   Akuammiline | -9.4 | -21.3 | -15.0 | -18.7 | -18.3 | -19.2 | -17.8 | -21.2 | -23.1 | -19.0 | -4.3 | -21.1 | -20.9 |
|   Akuammine | -14.7 | -19.8 | -17.8 | -16.9 | -19.6 | -17.4 | -15.6 | -20.1 | -21.5 | -17.6 | -6.7 | -19.7 | -21.8 |
|   Burnamine | -7.1 | -19.3 | -18.9 | -19.5 | -18.9 | -19.5 | -16.5 | -19.9 | -19.0 | -18.6 | -3.1 | -22.1 | -20.8 |
|   *Picralima nitida* coumestan **1** | -30.1 | -34.9 | -35.9 | -37.7 | -32.6 | -28.2 | -32.0 | **-38.6** | -34.7 | -28.9 | -21.3 | **-40.2** | -34.0 |
|   *Picralima nitida* coumestan **2** | -33.1 | **-44.1** | -38.5 | -38.7 | -32.9 | -28.6 | -34.1 | -37.4 | -37.0 | -26.4 | -11.6 | **-43.7** | -37.4 |
|   *Picralima nitida* coumestan **3** | -29.5 | **-42.7** | -32.5 | -35.0 | -33.6 | -31.8 | -31.9 | -38.8 | -37.1 | -29.1 | -7.8 | -38.9 | -38.5 |
|   *Picralima nitida* coumestan **4** | -26.9 | -32.9 | **-34.0** | -28.2 | -29.9 | -23.1 | -27.1 | -30.1 | -30.6 | -28.1 | -28.0 | **-34.1** | -31.1 |
|   *Picralima nitida* coumestan **5** | -26.6 | -33.4 | **-34.9** | -29.1 | -28.4 | -23.5 | -27.9 | -31.9 | -32.4 | -28.6 | -26.0 | **-35.4** | -32.1 |
|   *Picralima nitida* coumestan **6** | -27.7 | **-35.7** | **-34.6** | -31.1 | -28.6 | -25.6 | -27.7 | -32.1 | -31.6 | -27.5 | -27.5 | **-34.0** | -34.1 |
|  |  |  |  |  |  |  |  |  |  |  |  |  |  |
|   Pericine | -16.1 | -21.4 | -20.1 | -21.4 | -21.4 | -16.6 | -17.7 | -19.9 | -20.8 | -19.7 | -20.5 | -21.2 | -20.6 |
|   Picralinal | -10.7 | -20.9 | -17.3 | -19.1 | -19.0 | -19.6 | -16.0 | -18.2 | **-21.9** | -17.9 | no dock | -16.4 | -20.2 |
|   Picraline | no dock | -23.2 | -21.6 | -22.3 | -20.8 | -20.1 | -18.7 | -22.2 | **-27.4** | -19.4 | -5.8 | -22.9 | -23.4 |
|   Picranitine | -11.2 | -20.0 | -15.2 | -18.8 | -18.0 | -18.6 | -16.9 | -22.5 | -19.1 | -17.8 | -5.1 | -22.4 | -22.0 |
|   Picraphylline | -21.7 | -25.0 | -20.3 | -25.3 | -23.5 | -22.3 | -22.1 | -23.2 | -25.1 | -23.2 | -19.5 | **-25.6** | -22.4 |
|   Picratidine | -13.8 | -24.0 | -18.6 | -22.2 | -20.4 | -19.6 | -16.9 | -23.1 | -15.5 | -15.9 | -4.7 | -21.9 | -24.3 |
|   Pseudoakuammigine | -13.4 | -18.6 | -16.8 | -15.2 | -17.2 | -16.0 | -16.2 | -20.1 | -20.5 | -14.9 | no dock | -20.0 | -20.7 |
|   Rhazimol | no dock | -17.8 | -17.1 | -19.8 | -18.4 | -17.3 | -15.2 | -16.5 | -18.6 | -18.5 | -2.5 | -20.6 | -18.2 |

^a^Ligands showing selective (significantly stronger docking than average for all proteins) docking energies are highlighted in **blue bold**.
