# Supplementary material for: In-silico Investigation of Antitrypanosomal Phytochemicals from Nigerian Medicinal Plants
Source: PLoS Negl Trop Dis. 2012 Jul 24;6(7):e1727. doi: 10.1371/journal.pntd.0001727 (PMC3404109; doi:10.1371/journal.pntd.0001727)
Supplement: Table S17 — Lowest-energy docking energies (kcal/mol) for Prosopis africana phytochemicals with Trypanosoma brucei protein targets. (DOCX) [file pntd.0001727.s017.docx]

**Table S17.** Lowest-energy docking energies (kcal/mol) for *Prosopis africana* phytochemicals with *Trypanosoma brucei* protein targets.^a^

| Compound | Rhodesain | TbAK | TbPTR1 | TbDHFR | TbTR | TbCatB | TbHSP90 | TbCYP51 | TbNH | TbTIM | TbNDRT | TbUDPGE | TbODC |
| --- | --- | --- | --- | --- | --- | --- | --- | --- | --- | --- | --- | --- | --- |
|   Prosafrine | -23.6 | **-26.2** | -25.4 | -23.8 | -23.5 | -21.6 | -24.6 | -24.2 | -24.7 | -24.8 | -25.9 | -26.8 | -25.3 |
|   Isoprosopinine A | -24.4 | **-27.6** | -27.1 | -24.9 | -24.1 | -21.1 | -24.5 | -26.0 | -25.5 | -24.1 | -27.4 | -28.3 | -24.0 |
|   Isoprosopinine B | -24.6 | **-27.4** | -25.3 | -23.7 | -22.9 | -21.5 | -26.8 | -24.6 | -26.6 | -22.1 | -26.7 | -28.4 | -25.3 |
|   Prosafrinine | -23.4 | -26.2 | -26.6 | -22.4 | -22.9 | -22.1 | -27.0 | -22.8 | -24.4 | -24.7 | -24.9 | -27.2 | -23.3 |
|   Prosopine | -24.6 | -26.2 | -25.8 | -26.4 | -25.1 | -22.0 | -25.3 | -25.0 | -24.5 | -24.9 | -25.7 | -26.7 | -23.2 |
|   Spectaline | -23.8 | **-27.7** | -26.9 | -24.4 | -23.2 | -21.1 | -25.2 | -25.5 | -25.3 | -23.6 | -25.2 | -29.5 | -23.8 |

^a^Ligands showing selective (significantly stronger docking than average for all proteins) docking energies are highlighted in **blue bold**.
