# Supplementary material for: In-silico Investigation of Antitrypanosomal Phytochemicals from Nigerian Medicinal Plants
Source: PLoS Negl Trop Dis. 2012 Jul 24;6(7):e1727. doi: 10.1371/journal.pntd.0001727 (PMC3404109; doi:10.1371/journal.pntd.0001727)
Supplement: Table S18 — Lowest-energy docking energies (kcal/mol) for Rauwolfia vomitoria phytochemicals with Trypanosoma brucei protein targets. (DOCX) [file pntd.0001727.s018.docx]

**Table S18.** Lowest-energy docking energies (kcal/mol) for *Rauwolfia vomitoria* phytochemicals with *Trypanosoma brucei* protein targets.^a^

| Compound | Rhodesain | TbAK | TbPTR1 | TbDHFR | TbTR | TbCatB | TbHSP90 | TbCYP51 | TbNH | TbTIM | TbNDRT | TbUDPGE | TbODC |
| --- | --- | --- | --- | --- | --- | --- | --- | --- | --- | --- | --- | --- | --- |
|   3-Epirescinnamine | -24.5 | **-35.7** | **-36.6** | -27.4 | -30.8 | -25.6 | -27.7 | -27.4 | -28.9 | -13.0 | no dock | -28.6 | **-36.2** |
|   3-Isoreserpine | -15.6 | **-36.4** | -30.1 | -30.9 | -28.5 | -25.8 | -29.0 | -28.8 | -27.8 | -22.5 | -7.2 | -30.8 | -30.5 |
|   10-Hydroxygeissoschizol | -20.7 | -25.4 | **-27.1** | -22.9 | -25.8 | -20.1 | -25.2 | -23.2 | -24.4 | -25.0 | -23.7 | **-27.5** | -24.9 |
|   10-Hydroxynortetraphyllicine | -10.0 | -21.2 | -21.0 | -18.6 | -23.5 | -14.1 | -16.4 | -19.3 | -21.2 | -19.0 | -14.9 | -24.1 | -18.3 |
|   10-Methoxygeissoschizol | -21.2 | -23.2 | **-27.4** | -22.5 | -26.3 | -20.3 | -23.2 | -23.6 | -26.2 | -25.5 | -24.1 | **-26.9** | -23.6 |
|   12-Hydroxyajmaline | -10.8 | -18.7 | -22.3 | -19.6 | -23.6 | -18.0 | -19.4 | -22.4 | -19.9 | -17.0 | -14.0 | -25.5 | -20.4 |
|   18-Hydroxyyohimbine | -21.0 | -25.4 | -25.5 | -24.0 | -24.7 | -23.3 | **-28.1** | -23.3 | -27.2 | -25.2 | -21.0 | -26.9 | -25.9 |
|   19,20-Dehydroreserpiline | -18.6 | -29.3 | -24.1 | -26.4 | -27.2 | -22.5 | -25.9 | -26.4 | -29.3 | -20.3 | -17.8 | **-32.9** | -26.2 |
|   Ajmalidine | -11.5 | -19.5 | -20.2 | -17.7 | -21.9 | -13.5 | -18.4 | -20.2 | -20.4 | -16.3 | -13.7 | -23.9 | -18.5 |
|   Ajmalimine | -20.1 | -27.7 | -27.4 | -24.2 | **-29.5** | -19.1 | -28.8 | -26.1 | **-29.1** | -21.9 | no dock | -21.4 | -24.5 |
|   Ajmaline | -10.9 | -18.1 | -20.7 | -17.2 | -22.2 | -17.2 | -17.9 | -21.1 | -20.6 | -17.0 | -13.5 | -24.4 | -18.3 |
|   Ajmalinol | -13.2 | -19.1 | -21.0 | -18.2 | -21.9 | -16.3 | -19.2 | -23.0 | -19.9 | -18.6 | -13.8 | -24.8 | -19.6 |
|   Carapanaubine | -22.2 | -26.4 | -25.2 | -23.6 | -24.4 | -22.3 | -22.4 | -25.4 | -17.3 | -25.0 | -22.4 | -26.6 | **-26.8** |
|   Carapanaubine-*N*-oxide | -22.7 | **-28.1** | -26.9 | -23.9 | -25.2 | -21.3 | -22.1 | -24.9 | -18.4 | -24.8 | -18.7 | -26.5 | -25.6 |
|   Geissoschizol | -20.3 | -24.6 | -25.7 | -20.9 | -25.0 | -19.4 | -23.2 | -20.8 | -24.2 | -23.3 | -23.1 | **-27.4** | -22.4 |
|   Isoajmaline | -11.8 | -17.3 | -21.6 | -18.5 | **-23.9** | -17.7 | -13.9 | -22.1 | -20.7 | -17.0 | -8.0 | -23.0 | -18.0 |
|   Isocarapanaubine | -18.0 | **-28.4** | -22.2 | -24.8 | -24.9 | -24.2 | -20.9 | -27.5 | -5.9 | -19.1 | -21.4 | -25.4 | -24.7 |
|   Isoreserpiline | -21.1 | -27.4 | **-29.5** | -23.6 | -26.9 | -21.4 | -23.5 | -26.8 | **-29.7** | -27.0 | -19.3 | -28.8 | -28.0 |
|   Isoreserpiline pseudoindoxyl 1 | -18.3 | -26.4 | -25.3 | -22.5 | -26.2 | -25.1 | -25.8 | -26.2 | -20.5 | -21.4 | -20.8 | **-28.2** | -25.9 |
|   Isosandwicine | -13.0 | -19.3 | -21.1 | -15.6 | -20.9 | -17.7 | -16.9 | -20.6 | -20.6 | -17.1 | -14.6 | -23.0 | -17.8 |
|   Methyl 3,4-dimethoxybenzoylreserpate | -18.4 | **-35.0** | -22.6 | -27.9 | -28.2 | -28.8 | -22.1 | -32.2 | -24.4 | -19.6 | -8.9 | **-37.6** | -31.9 |
|  |  |  |  |  |  |  |  |  |  |  |  |  |  |
|   Methyl reserpate | -19.6 | -26.2 | -23.0 | -26.2 | -23.5 | -20.7 | -22.5 | -24.2 | -27.5 | -21.3 | -12.9 | **-32.5** | -24.2 |
|   Mitoridine | -14.2 | -18.0 | -21.7 | -19.1 | **-23.5** | -15.7 | -18.2 | -20.7 | **-22.4** | -17.0 | -14.9 | -22.5 | -19.2 |
|   Neonorreserpine | -17.8 | **-34.6** | -26.4 | -30.4 | -28.5 | -26.8 | -29.4 | **-34.7** | -26.4 | -27.0 | -6.4 | -29.9 | -31.1 |
|   Normitoridine | -15.3 | **-22.5** | -20.5 | -18.8 | **-22.5** | -14.7 | -18.2 | -20.7 | -21.3 | -20.4 | -14.6 | -21.3 | -17.9 |
|   Norpurpeline | -14.0 | **-23.4** | -20.4 | -17.2 | -22.3 | -13.3 | -19.1 | -22.3 | -21.9 | -22.0 | -16.3 | -23.0 | -19.3 |
|   Norrauvomitine | -20.1 | -28.5 | -25.7 | -27.1 | -25.2 | -18.3 | -25.5 | -26.0 | -29.0 | -20.5 | -1.2 | -24.8 | -26.6 |
|   Norseredamine | -15.3 | -20.4 | -21.1 | -18.1 | -22.7 | -16.5 | -18.7 | -21.7 | -22.0 | -21.4 | -17.1 | -24.0 | -19.2 |
|   Nortetraphyllicine | -15.2 | -19.0 | -20.9 | -16.5 | **-22.4** | -15.7 | -16.9 | -20.1 | -20.4 | -18.3 | -14.0 | -22.3 | -16.3 |
|   Perakine | -14.5 | -21.8 | -23.5 | -20.6 | -23.1 | -18.9 | -19.4 | -20.1 | **-24.4** | -17.9 | -13.1 | -21.6 | -19.2 |
|   Picrinine | -13.0 | -20.3 | -21.4 | **-23.7** | -21.0 | -17.5 | -18.7 | -21.2 | -18.6 | -21.4 | -16.7 | -22.4 | -19.8 |
|   Purpeline | -12.4 | -18.6 | -22.4 | -16.8 | -21.6 | -16.0 | -18.7 | -21.1 | -22.4 | -17.1 | -15.8 | **-23.6** | -17.7 |
|   Raucaffrinoline | -14.1 | -20.5 | **-23.1** | -19.8 | **-23.8** | -19.1 | -19.3 | -20.2 | -22.5 | -18.9 | -15.3 | -22.1 | -19.1 |
|   Raumitorine | -23.2 | -27.8 | **-29.9** | -24.9 | -27.2 | -21.4 | -25.1 | -23.5 | -26.4 | -25.9 | -17.3 | -27.4 | -27.2 |
|   Rauvanine | -24.1 | -28.7 | **-30.0** | -22.4 | -26.1 | -18.9 | -26.9 | -25.6 | -26.5 | -25.4 | -18.6 | -28.3 | -26.3 |
|   Rauvomitine | -18.8 | -27.7 | -26.7 | -25.1 | -26.5 | -17.7 | -27.3 | -25.6 | **-29.6** | -22.4 | no dock | -24.6 | -25.4 |
|   Rauvoxine | -21.6 | **-27.2** | -23.9 | -24.4 | -24.9 | -22.2 | -21.4 | -24.4 | -19.6 | -24.9 | -15.8 | -23.8 | **-26.2** |
|   Rauvoxinine | -24.2 | -26.4 | -26.0 | -24.1 | -25.9 | -19.9 | -24.2 | **-27.0** | -15.4 | -21.7 | -21.6 | -25.2 | -21.9 |
|   Renoxydine | -20.7 | -29.5 | -26.0 | -29.1 | -25.8 | -27.1 | -25.8 | **-35.1** | -21.3 | -22.4 | no dock | **-36.2** | -32.4 |
|   Rescidine | -19.8 | -33.5 | -26.8 | -24.6 | -29.7 | -29.5 | -28.3 | **-39.1** | -23.8 | -25.6 | -11.7 | **-36.1** | -31.0 |
|   Rescinnamine | -22.7 | -33.7 | -31.8 | -24.0 | -28.1 | -27.4 | -26.1 | -32.6 | -23.2 | -19.1 | -7.5 | **-37.1** | -34.7 |
|   Reserpic acid | -21.9 | -26.2 | -20.9 | -25.9 | -22.3 | -22.7 | -21.4 | -23.1 | -26.0 | -20.7 | -14.5 | **-30.5** | -24.0 |
|   Reserpiline | -17.5 | -28.5 | -24.2 | -26.1 | -23.2 | -21.1 | -25.4 | -26.5 | -29.0 | -20.0 | -20.6 | **-33.1** | -25.6 |
|   Reserpine | -23.5 | -29.4 | -28.0 | -26.3 | -29.5 | -26.3 | -27.0 | **-34.0** | -16.4 | -21.4 | -12.6 | **-35.7** | -31.1 |
|   Reserpinine oxindole | -18.9 | -25.5 | -25.0 | -23.8 | -25.6 | -23.3 | -23.6 | -25.4 | -19.9 | -22.6 | -24.3 | **-27.5** | -25.5 |
|   Sandwicine | -13.0 | -19.3 | -19.8 | -16.8 | -20.4 | -16.7 | -18.2 | -19.7 | -19.6 | -15.7 | -13.9 | -21.9 | -18.2 |
|   Seredamine | -16.0 | -19.5 | -22.8 | -17.5 | -22.8 | -15.0 | -18.2 | -21.9 | -21.7 | -18.1 | -13.9 | **-23.3** | -16.7 |
|   Seredine | -18.9 | -27.1 | -30.6 | -22.9 | -27.7 | -18.9 | -27.4 | -28.2 | -30.5 | -24.0 | -10.3 | -29.9 | -27.8 |
|   Serpenticine | -19.9 | -25.6 | -25.7 | -22.0 | -24.2 | -17.9 | -25.0 | -23.8 | **-28.4** | -23.3 | -21.6 | -26.6 | -25.0 |
|  |  |  |  |  |  |  |  |  |  |  |  |  |  |
|   Strictamine | -14.0 | -19.8 | -19.7 | -21.4 | -19.6 | -18.3 | -17.8 | -20.2 | -17.4 | -18.3 | -3.7 | -21.1 | -18.5 |
|   Tetraphyllicine | -12.3 | -17.9 | -20.9 | -17.5 | -21.7 | -14.9 | -17.4 | -20.4 | -20.7 | -16.5 | -11.9 | **-22.9** | -17.2 |
|   Tombozine | -13.4 | -19.8 | -23.1 | -19.1 | -21.5 | -17.6 | -18.7 | -20.3 | -21.9 | **-23.7** | -20.5 | **-25.1** | -19.8 |
|   Vellosimine | -13.5 | -21.4 | -23.1 | -19.3 | -21.2 | -17.7 | -18.1 | -19.9 | -22.0 | **-23.8** | -20.7 | **-24.5** | -18.9 |
|   Vomalidine | -12.8 | -21.4 | -22.1 | -18.5 | -22.6 | -15.6 | -19.7 | -20.9 | -21.3 | -19.3 | -15.5 | **-27.1** | -19.5 |
|   Vomifoliol | -16.2 | -19.0 | -19.2 | -17.4 | -19.3 | -14.4 | -16.4 | -19.9 | -19.1 | -15.9 | -18.0 | -19.4 | -17.5 |
|   Vomilenine | -13.1 | -19.9 | -22.4 | -21.3 | -22.6 | -17.1 | -18.9 | -21.5 | -22.9 | -18.2 | -12.8 | -22.0 | -21.0 |

^a^Ligands showing selective (significantly stronger docking than average for all proteins) docking energies are highlighted in **blue bold**.
