# Supplementary material for: In-silico Investigation of Antitrypanosomal Phytochemicals from Nigerian Medicinal Plants
Source: PLoS Negl Trop Dis. 2012 Jul 24;6(7):e1727. doi: 10.1371/journal.pntd.0001727 (PMC3404109; doi:10.1371/journal.pntd.0001727)
Supplement: Table S19 — Lowest-energy docking energies (kcal/mol) for Securidaca longipedunculata phytochemicals with Trypanosoma brucei protein targets. (DOCX) [file pntd.0001727.s019.docx]

**Table S19.** Lowest-energy docking energies (kcal/mol) for *Securidaca longipedunculata* phytochemicals with *Trypanosoma brucei* protein targets.^a^

| Compound | Rhodesain | TbAK | TbPTR1 | TbDHFR | TbTR | TbCatB | TbHSP90 | TbCYP51 | TbNH | TbTIM | TbNDRT | TbUDPGE | TbODC |
| --- | --- | --- | --- | --- | --- | --- | --- | --- | --- | --- | --- | --- | --- |
|   (*E*)-Ethyl 4-methoxycinnamate | -19.6 | -20.1 | -20.1 | -20.3 | -19.2 | -15.6 | -17.8 | -19.4 | -19.8 | **-21.8** | -18.5 | -20.3 | -19.4 |
|   (*E*)-Methyl 3,4-dimethoxycinnamate | -19.0 | -21.2 | -21.1 | -20.9 | -19.2 | -17.0 | -19.5 | -20.6 | -20.9 | **-22.5** | -19.8 | -22.4 | -20.4 |
|   (*E*)-Methyl 3,4,5-trimethoxycinnamate | -19.6 | -22.3 | -22.7 | -21.5 | -20.7 | -19.2 | -20.7 | -21.1 | -21.9 | -22.4 | -21.1 | **-24.7** | -19.8 |
|   (*E*)-Methyl sinapate | -19.4 | -22.0 | -22.3 | -21.7 | -20.0 | -17.7 | -21.5 | -19.0 | -21.7 | -21.9 | -20.6 | **-23.7** | -20.9 |
|   (*Z*)-Ethyl 4-methoxycinnamate | -16.8 | -18.7 | -18.9 | -17.6 | -18.9 | -13.8 | -19.2 | -17.1 | -18.4 | **-20.4** | -18.3 | -19.1 | -18.3 |
|   (*Z*)-Methyl 3,4-dimethoxycinnamate | -17.9 | -19.3 | -20.9 | -19.5 | -19.4 | -16.1 | -21.0 | -18.3 | -19.8 | **-22.1** | -20.4 | -21.7 | -21.0 |
|   (*Z*)-Methyl 4-methoxycinnamate | -16.8 | -18.6 | -19.6 | -17.7 | -18.7 | -13.4 | -19.5 | -18.1 | -18.6 | **-20.5** | -18.2 | -19.5 | -18.7 |
|   1,3,6,8-Tetrahydroxy-2,5-dimethoxyxanthone | -20.0 | -22.7 | **-25.0** | -20.2 | -22.8 | -16.7 | -22.2 | -20.1 | -23.5 | -24.1 | -19.5 | **-25.8** | -21.8 |
|   1,6,8-Trihydroxy-2,3,4,7-tetramethoxyxanthone | -19.8 | -25.3 | -24.3 | -19.2 | -21.4 | -16.4 | -21.2 | -23.6 | -24.3 | -25.1 | -19.6 | **-27.1** | -22.5 |
|   2-Hydroxy-1,7-dimethoxyxanthone | -17.8 | -21.0 | **-22.4** | -18.3 | -20.5 | -18.7 | -20.9 | -18.4 | -20.5 | -19.9 | -18.4 | **-23.7** | -19.6 |
|   Dehydroelymoclavine | -18.2 | -21.7 | **-23.4** | -18.0 | -22.5 | -15.3 | -21.4 | -19.9 | -20.7 | -22.6 | -19.0 | **-23.9** | -20.2 |
|   *S. longipedunculata* alkaloid A | -19.2 | **-27.5** | -23.7 | -22.3 | -22.0 | -19.1 | -24.2 | -23.9 | -25.5 | -26.1 | -17.9 | **-29.1** | -24.4 |

^a^Ligands showing selective (significantly stronger docking than average for all proteins) docking energies are highlighted in **blue bold**.
