# Supplementary material for: In-silico Investigation of Antitrypanosomal Phytochemicals from Nigerian Medicinal Plants
Source: PLoS Negl Trop Dis. 2012 Jul 24;6(7):e1727. doi: 10.1371/journal.pntd.0001727 (PMC3404109; doi:10.1371/journal.pntd.0001727)
Supplement: Table S20 — Lowest-energy docking energies (kcal/mol) for Strychnos spinosa phytochemicals with Trypanosoma brucei protein targets. (DOCX) [file pntd.0001727.s020.docx]

**Table S20.** Lowest-energy docking energies (kcal/mol) for *Strychnos spinosa* phytochemicals with *Trypanosoma brucei* protein targets.^a^

| Compound | Rhodesain | TbAK | TbPTR1 | TbDHFR | TbTR | TbCatB | TbHSP90 | TbCYP51 | TbNH | TbTIM | TbNDRT | TbUDPGE | TbODC |
| --- | --- | --- | --- | --- | --- | --- | --- | --- | --- | --- | --- | --- | --- |
|   10-Hydroxyakagerine | -20.4 | -25.0 | **-28.4** | -22.2 | -22.0 | -21.4 | -23.9 | -23.0 | -25.5 | -23.2 | -16.6 | **-27.2** | -24.3 |
|   3-(1-Hydroxyethyl)-5-methoxycarbonylpyridine | -15.7 | -17.6 | -19.1 | -16.0 | -17.0 | -14.1 | -18.6 | -17.5 | -17.3 | **-19.5** | -16.9 | -18.1 | -18.0 |
|   3-(1-Hydroxyethyl)-4-methyl-5-methoxycarbonylpyridine | -15.7 | -17.7 | -19.4 | -16.3 | -16.8 | -14.2 | -19.1 | -18.0 | -16.4 | **-21.3** | -16.3 | -19.1 | -17.2 |
|   5-Methoxycarbonyl-4-methyl-2,7-naphthyridine | -15.1 | -18.1 | **-20.0** | -16.9 | -18.2 | -13.7 | -18.7 | -17.1 | -16.7 | **-20.0** | -15.9 | -19.0 | -16.7 |
|   6-*O*-Nicotinoyltetrahydrocantleyine | -18.8 | -27.2 | **-27.7** | -22.0 | -23.7 | -19.2 | -25.4 | -23.8 | -25.0 | -25.2 | -25.5 | -25.8 | -22.8 |
|   24-Hydroperoxy-24-vinylcholesterol | -6.0 | -26.2 | -25.2 | -22.4 | -25.9 | -20.7 | -22.5 | **-28.0** | -27.5 | -26.7 | -22.4 | -27.9 | **-28.0** |
|   Akagerine | -20.4 | -23.7 | **-26.6** | -21.4 | -23.8 | -19.7 | -23.8 | -21.1 | -23.8 | -22.2 | -17.8 | -26.0 | -21.9 |
|   α-Amyrin | -5.7 | -21.7 | -13.1 | -20.3 | -16.9 | -16.6 | -15.8 | **-23.8** | -15.5 | -17.0 | -10.5 | -22.4 | -22.2 |
|   β-Amyrin | no dock | -20.8 | -13.9 | -19.8 | -14.3 | -16.4 | -14.9 | **-23.6** | -16.7 | -13.6 | -14.9 | -20.1 | **-24.6** |
|   Betulin | -2.4 | -22.0 | -19.6 | -18.5 | -20.0 | -19.1 | -15.1 | **-25.1** | -20.4 | -15.5 | -10.2 | -17.7 | **-24.9** |
|   Betulinic acid | -11.3 | -22.1 | -19.9 | -14.6 | -21.0 | -15.5 | -18.4 | **-25.8** | -20.6 | -15.1 | -12.9 | -20.7 | **-25.3** |
|   Clerosterol | -15.9 | -24.2 | -24.6 | -23.3 | -24.0 | -20.5 | -23.9 | **-28.6** | -27.8 | -26.0 | -21.6 | **-28.2** | -26.9 |
|   Erythrodiol | -13.5 | -21.5 | -11.0 | -19.8 | -16.8 | -16.0 | -15.9 | **-24.6** | -17.1 | -9.1 | -16.5 | -23.8 | **-24.9** |
|   Henningsoline | -17.4 | **-24.6** | -16.9 | -20.9 | -22.1 | -19.8 | -21.1 | -23.5 | -18.4 | -21.6 | -17.7 | -23.4 | -21.5 |
|   Kingiside aglucone | -15.6 | -19.0 | -19.5 | -16.2 | -19.3 | -15.4 | **-21.3** | -19.7 | -19.0 | -17.2 | -18.4 | -20.0 | -16.4 |
|   Kribine | -19.3 | -25.0 | **-27.7** | -21.1 | -23.8 | -16.4 | -20.7 | -19.6 | -23.8 | -24.9 | -16.1 | -24.3 | -20.5 |
|   Lupeol | -11.3 | -21.5 | -18.5 | -17.4 | -19.4 | -18.5 | -17.1 | **-24.3** | -19.7 | -15.2 | -8.1 | -19.0 | **-24.9** |
|   Neozeylanicine | -15.2 | -18.4 | -19.8 | -16.9 | -18.3 | -14.1 | -19.5 | -17.6 | -17.6 | **-20.6** | -16.0 | -19.8 | -17.7 |
|   Scaevodimerine A | -22.8 | -27.1 | -26.8 | -28.3 | -25.1 | -21.0 | -28.7 | -24.7 | -25.9 | -24.1 | -26.4 | **-30.4** | -27.5 |
|   Seringosterol | -16.4 | -27.2 | -25.2 | -24.4 | -24.5 | -21.1 | -22.2 | -27.7 | -27.6 | -26.8 | -21.8 | -29.2 | **-28.5** |
|   β-Sitosterol | -18.2 | -24.6 | -24.4 | -23.7 | -23.8 | -20.3 | -20.4 | **-28.6** | -26.8 | -27.1 | -22.3 | **-29.7** | -26.5 |
|   Stigmasterol | -15.2 | -25.2 | -23.5 | -24.1 | -24.3 | -19.1 | -22.7 | -28.3 | -26.9 | -27.5 | -21.5 | **-29.6** | -26.6 |
|   Strychnoside A | -23.3 | **-39.8** | -30.3 | -27.6 | -30.9 | -26.0 | -32.1 | -34.4 | -33.3 | -17.0 | -15.4 | **-37.2** | -35.5 |
|   Stryspinolactone | -16.6 | -19.3 | **-21.1** | -18.5 | **-21.1** | -17.1 | -19.4 | -18.7 | -21.2 | -20.4 | -18.0 | **-22.1** | -17.6 |
|   Stryspinoside | -20.6 | -28.7 | -25.5 | -24.9 | -25.7 | -22.2 | -26.7 | -29.3 | -27.4 | -26.8 | -18.7 | **-30.2** | -26.2 |
|   Stryspinoside aglucone | -13.3 | -22.2 | -21.9 | -18.2 | -20.3 | -16.9 | -16.4 | **-23.0** | **-23.5** | -10.2 | -16.4 | -22.8 | -16.8 |
|  |  |  |  |  |  |  |  |  |  |  |  |  |  |
|   Uvaol | -13.5 | -22.6 | -9.8 | -20.7 | -19.7 | -17.6 | -16.2 | **-25.0** | -17.5 | -9.1 | -8.0 | -23.8 | -22.5 |

^a^Ligands showing selective (significantly stronger docking than average for all proteins) docking energies are highlighted in **blue bold**.
